# Synthesis and Biological Evaluation of 5′-*O*-Fatty Acyl Ester Derivatives of 3′-Fluoro-2′,3′-dideoxythymidine as Potential Anti-HIV Microbicides

**DOI:** 10.3390/molecules27103352

**Published:** 2022-05-23

**Authors:** Hitesh K. Agarwal, Bhupender S. Chhikara, Guofeng Ye, Sitaram Bhavaraju, Ajay Dixit, Anil Kumar, Gustavo F. Doncel, Keykavous Parang

**Affiliations:** 1 Department of Pharmaceutical Sciences, School of Pharmacy, South University, 709 Mall Boulevard, Savannah, GA 31406, USA; 2 Department of Biomedical and Pharmaceutical Sciences, School of Pharmacy, University of Rhode Island, Kingston, RI 02881, USA; bschhikara@gmail.com (B.S.C.); guofengye@gmail.com (G.Y.); sitaramb4u@hotmail.com (S.B.); dixitak2000@yahoo.com (A.D.); anilkadian@gmail.com (A.K.); 3 ITC Life Science & Technology Center, #3, 1st Main, Peenya Industrial Area, 1st Phase, Bangalore 560058, India; 4 CONRAD, Department of Obstetrics and Gynecology, Eastern Virginia Medical School, Norfolk, VA 23507, USA; 5 Center for Targeted Drug Delivery, Department of Biomedical and Pharmaceutical Sciences, Chapman University School of Pharmacy, Harry and Diane Rinker Health Science Campus, Irvine, CA 92618, USA

**Keywords:** anti-HIV, cellular uptake, cytotoxicity, fatty acids, 3′-fluoro-2′,3′-dideoxythymidine, multidrug-resistant, proinflammatory cytokine

## Abstract

A number of 5′-*O*-fatty acyl derivatives of 3′-fluoro-2′,3′-dideoxythymidine (FLT, **1**) were synthesized. These conjugates were evaluated for their potential as topical microbicides with anti-HIV activity against cell-free (X4 and R5), cell-associated, and multidrug-resistant viruses. Compared to FLT and 3′-azido-2′,3′-dideoxythymidine (AZT), 5′-*O*-(12-azidododecanoyl) (**5**), 5′-*O*-myristoyl (**6**), and 5′-*O*-(12-thioethyldodecanoyl) (**8**) derivatives of FLT were found to be more active against both cell-free viruses (lymphocytotropic and monocytotropic strains) with EC_50_ values of 0.4 μM, 1.1 μM, and <0.2 μM, respectively, as well as cell-associated virus with EC_50_ values of 12.6, 6.4, and 2.3 μM, respectively. Conjugates **5**, **6**, and **8** exhibited >4 and >30 times better antiviral index than FLT and AZT, respectively. Conjugates **5** and **8** were significantly more potent than FLT against many multidrug-resistant strains. A comparison of the anti-HIV activity with the corresponding non-hydrolyzable ether conjugates suggested that ester hydrolysis to FLT and fatty acids is critical to enable anti-HIV activity. Cellular uptake studies were conducted using fluorescent derivatives of FLT attached with 5(6)-carboxyfluorescein through either β-alanine (**23**) or 12-aminododecanoic acid (**24**) spacers. The lipophilic fluorescent analog with a long chain (**24**) showed more than 12 times higher cellular uptake profile than the fluorescent analog with a short chain (**23**). These studies further confirmed that the attachment of fatty acids improved the cellular uptake of nucleoside conjugates. In addition, **5**, **6**, and **8** were the least cytotoxic and did not alter vaginal cell and sperm viability compared to the positive control, a commercial topical spermicide (N-9), which significantly decreased sperm and vaginal cell viability inducing the generation of proinflammatory cytokines.

## 1. Introduction

In June 1981, the very first case of acquired immunodeficiency syndrome (AIDS) in the United States was identified to be caused by the human immunodeficiency virus (HIV) [1]. For the next several years, AIDS was considered as an untreatable condition, tagging it as a death sentence. Today, HIV/AIDS has claimed more than 36 million lives worldwide according to UNAIDS 2022 Fact Sheet.

Significant research across the world resulted in the development of antiretroviral drugs for HIV. Using Highly Active Anti-Retroviral Therapy (HAART) on a daily basis, the viral content in an HIV-positive patient can be reduced to an undetectable level of HIV, which in turn significantly reduces transmission [2,3,4]. The global fight against HIV has been very effective; however, HIV is still slowly spreading. Recent experiences have taught us the value of protecting ourselves from other deadly viruses [5,6]. With 38 million people globally living with HIV and >1.7 million newly infected HIV cases reported in 2019, the transmission of HIV infection cannot be ignored [7]. Preventing viral spread will play a crucial role if the Sustainable Development Goal (United Nations) of ending the AIDS epidemic is to be met by the targeted deadline of 2030 [7,8]. Therefore, we are committed to the design and evaluation of fatty acid esters of nucleoside analogs for topical application and anti-HIV microbicides to prevent viral transmission. Microbicides are compounds that are topically applied to the vagina or rectum and may confer protection against sexually transmitted diseases including HIV.

We have previously reported a number of 5′-*O*-fatty acyl derivatives of alovudine (FLT, 3′-fluoro-2′,3′-dideoxythymidine, **1**) and Zidovudine (AZT, 3′-azido-2′,3′-dideoxythymidine) that exhibited a better and wider activity profile than FLT and AZT, respectively [9,10]. Among these, three FLT conjugates of myristic acid, 12-azidododecanoic acid, and 13-thiapentadecanoic acid provided promising results against the X4 and R5 strains of HIV through the synergistic inhibition of both cell-free virus and cell-associated virus.

We hypothesized that the attachment of nucleoside analogs to the long-chain myristic acid analogs enhances their lipophilicity and thus their cellular uptake. Once the conjugate enters the cells, it is expected to be hydrolyzed by esterase and generate two active molecules: (i) a nucleoside analog, targeting reverse transcriptase (RT), and (ii) a fatty acid targeting *N*-myristoyl transferase (NMT) enzymes. Nucleoside analogs are among the first class of drugs approved to treat HIV through RT inhibition. In addition, fatty acid analogs moderately contribute to the inhibition of NMT, which is involved in catalyzing the myristoylation of several proteins in the HIV life cycle (e.g., capsid protein p17, Pr160^gag-pol^, Pr55^gag^, p27^nef^) [11,12]. For example, 12-thioethyldodecanoic acid was moderately active (EC_50_ = 9.4 μM) against HIV-infected T4 lymphocytes [13]. Using these three fatty acids, we synthesized several ester conjugates of anti-HIV reverse transcriptase inhibitors (NRTIs) such as 2′,3′-didehydro-2′,3′-dideoxythymidine (stavudine, d4T), 2′,3′-dideoxy-3′-thiacytidine (lamivudine, 3TC), and 2′,3′-dideoxy-5-fluoro-3′-thiacytidine (emtricitabine, FTC), which showed enhanced activity against X4, R5, cell-associated, and/or multidrug-resistant virus when compared to their parent nucleosides [14,15,16,17].

While FLT displays similar pharmacokinetic parameters to AZT, it is significantly more potent than AZT [18,19,20]. FLT was under clinical evaluation from 1990 to 1992. The studies were stopped after FLT failed phase II clinical trials because of the observed hematological toxicities including neutropenia, leucopenia, and anemia [21,22,23]. A number of phosphoramidates and phosphorodiamidates of FLT have also been synthesized and evaluated, showing sub-micromolar inhibition of HIV-1 and HIV-2 replication and activity in the thymidine kinase-negative cell models [24]. There has been a resurgent interest in studies using ^18^F-FLT in PET imaging for cancer detection [25,26,27]. We have also previously shown that the *N*-myristoylglutamic acid derivative of FLT generated an organogel that could be used in the design of microbicidal gels [28]. Thus, we evaluated the fatty acyl ester of FLT as alternative anti-HIV microbicidal agents and preventive tools to avoid extensive in vivo systemic toxicity associated with several nucleoside analogs.

As described above, the replication of HIV-1 can be inhibited by heteroatom-containing analogs of myristic acid without accompanying cellular toxicity [29,30]. It has been previously reported that several fatty acids such as 2-methoxydodecanoic acid, 4-oxatetradecanoic acid, and 12-thioethyldodecanoic acid reduce HIV-1 replication in acutely infected T-lymphocytes. At *N*-terminal glycine, viral proteins (gag and nef) are covalently attached to myristic acid in the presence of NMT. Myristic acid attachment makes the proteins more hydrophobic, which improves the protein–protein and protein–membrane interactions [31]. For example, after the *N*-myristoylation, the p17 protein localizes itself toward the cell membrane, where a new virus is produced [32].

Herein, we report on the synthesis of additional fatty acyl ester conjugates of FLT (Table 1), a more extensive evaluation of their biological activities and a comparison with the AZT conjugates, and the cellular uptake of representative compounds. We further propose their potential use as anti-HIV preventative agents and microbicides.

## 2. Results and Discussion

### 2.1. Chemistry

**5′-*O*-(Fatty acyl) ester derivatives of FLT and AZT.** FLT was synthesized using thymidine as the starting material according to the previously reported procedure [33]. 5′-*O*-(Fatty acyl) ester derivatives of FLT and AZT (Table 1) were synthesized from the reaction of FLT or AZT with the corresponding fatty acids in the presence of oxalyl chloride and DMAP as described previously [9,10] at the scale of 100 mg. Compounds **5**, **6**, and **8** showed higher potency and minimal cellular toxicity when compared to the other compounds (Table 2). Thus, compounds **5** and **8** were then synthesized on a larger scale (25 g) for further biological evaluation, preclinical, and formulation studies. The compounds were purified first by using silica gel column chromatography and then preparative HPLC to achieve >99% purity.

**Scheme 1:**
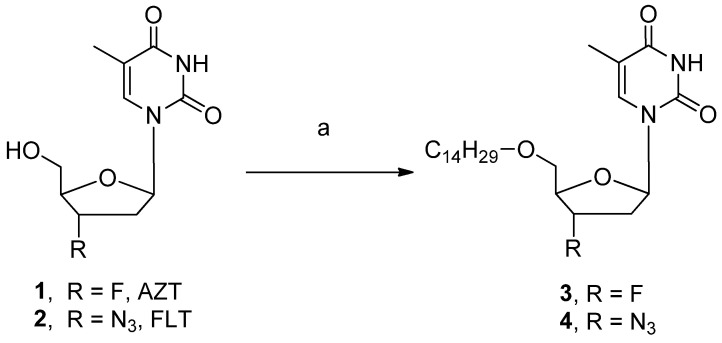
Synthesis of 5′-*O*-(tetradecanol) ether derivatives of FLT (**3**) and AZT (**4**). (a) Tetradecanol, PPh_3_, DIAD, DMF, 5 h, rt.

**5′-*O*-(Tetradecanol) ether derivatives of AZT and FLT.** AZT and FLT were reacted with tetradecanol in the presence of triphenylphosphine (TPP) and diisopropyl azodicarboxylate (DIAD) using Mitsunobu conditions to afford 5′-*O*-(tetradecanol) ether derivatives of FLT (**3**) and AZT and (**4**) (Scheme 1).

**Scheme 2:**
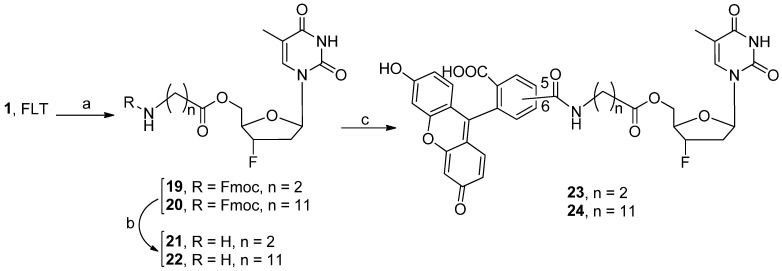
Synthesis of 5′-carboxyfluorescein derivatives of FLT (**23** and **24**). (a) Fmoc-amino acid, HBTU, DIPEA, DMF, 18 h, rt. (b) Piperidine, octanethiol, rt. (c) FAM, HBTU, DIPEA, DMR, 18 h, rt.

**5(6)-Carboxyfluorescein derivatives of FLT.** FLT was attached to 5(6)-carboxyfluorescein (FAM) using β-alanine and 12-aminododecanoic acid as linkers. FLT was esterified with the corresponding Fmoc-amino acids in the presence of HBTU and DIPEA, resulting in intermediates (**19**–**20**) followed by piperidine-assisted Fmoc deprotection to yield **21**–**22**. 5(6)-Carboxyfluorescein was conjugated at the free amino group in **21** and **22** in the presence of HBTU and DIPEA to produce 5(6)-carboxyfluorescein derivatives of FLT (Scheme 2, **23**–**24**). These compounds were used for the cellular uptake studies to determine the cellular uptake profile of the fatty acyl ester derivatives of FLT. FLT attached to FAM through β-alanine (**23**) was used as a control FLT analog. FLT attached to FAM through 12-aminododecanoyl (**24**) was used as an analog of 5′-*O*-(12-azidododecanoyl)-3′-fluoro-2′,3′-dideoxythymidine (**5**), and other fatty acid ester analogs of FLT.

### 2.2. Physicochemical Properties of ***5***

Physicochemical properties including pKa, Log D, and solubility were determined for **5** (as a model compound). The pKa of 9.67 was determined using dip-probe absorption spectroscopy (D-PAS technique) by Yasuda–Shedlovsky extrapolation of the dataset. It was not possible to measure the partition coefficient (Log P) of the fatty acyl derivatives of FLT and AZT in a standard n-octanol/water mixture because of the insolubility of the compounds in water. The cLog P value was calculated as 5.23 using ChemDraw. More accurately, the value of Log D of 5.04 at pH 7.4 was obtained by the liquid–liquid distribution chromatography method. Since most of **5** is in its neutral form, the Log D value will correspond to the Log P value.

The upper limit of aqueous solubility for **5** was determined to be 510 nM by the shake-flask methodology with UV-spectroscopic sample detection. Conjugate **5** was completely soluble in ethanol (>30 mg/mL) and the mixture of water/methanol (60:40), but slightly less soluble in DMSO (~4.1 mg/mL). These results indicate that the conjugates are very hydrophobic and are less likely to be ionized at physiological pH.

### 2.3. Biological Activities

#### 2.3.1. Anti-HIV Activities against Cell-Free and Cell-Associated Virus

Table 2, Table 3 and Table 4 illustrate the anti-HIV-1 activities against the cell-free, cell-associated, and multidrug-resistant virus and sperm inhibiting properties of the selected compounds. In summary, the anti-HIV activity of 5′-substituted derivatives of FLT and AZT (**5**–**18**) was dependent on the nature of the 5′-substituent. Similar to the parent nucleosides, the 5′-*O*-fatty acyl derivatives of FLT exhibited higher anti-HIV activities against HIV compared to the AZT derivatives in a single-round infection assay (Table 2). Among the FLT analogs, **5**, **6, 8**, and **13** derivatives had significant anti-HIV activity at noncytotoxic concentrations. As shown in Table 2, compounds **5**, **6**, and **8** displayed EC_50_ values against cell-free X4 and R5 viruses equal or less than 1 μM. Additionally, **5**, **6**, and **8** were also active against cell-associated HIV with EC_50_ values between 2.3 and 12.6 μM. None of the other esters were active against cell-associated virus. On the other hand, under the conditions employed to assess this activity, FLT and AZT were only inconsistently and moderately active against the cell-associated virus. The difference in the activity of fatty acyl derivatives of FLT compared to each other and FLT may be due to their differences in the rate of uptake and intracellular hydrolysis.

By design, the ester moiety in the conjugates needs to be hydrolyzed to render parent nucleosides and fatty acids to produce anti-HIV activity. The anti-HIV activities of 5′-*O*-ether substituted FLT and AZT (**3** and **4**, Scheme 2) with a stable tetradecanyl (myristyl) group were compared with hydrolyzable 5′-*O*-ester substituted FLT and AZT (**6** and **7**). Both ether derivatives (**3** and **4**) were significantly less potent than the corresponding ester derivatives (**6** and **7**). These data demonstrate that the ester bonds are important in enabling the anti-HIV activity of the fatty acyl ester derivatives of FLT and AZT (**5**–**18**). Results indicated that the anti-HIV activity of these conjugates were not due to their incorporation in the HIV membrane and/or to a detergent effect, as lipophilic ether derivatives, **3** and **4** were not potent anti-HIV agents.

Table 3 displays the anti-HIV activity of FLT or AZT, their equimolar mixture with fatty acids (**25**–**27**), and selected ester conjugates (**6**, **7**, and **12**). All ester conjugates were more active than their corresponding physical mixtures. The 5′-*O*-myristoyl FLT (**6**) was more active and consistent in displaying antiviral activity against the cell-associated virus than FLT and its physical mixture with myristic acid (**25**) (myristic acid and FLT (50:50). This physical mixture of myristic acid and FLT (**25**, EC_50_ = 33 μM) exhibited a 5.2-times lower potency against the cell-associated virus than (**6**, EC_50_ = 6.4 μM), suggesting that conjugation is required for the efficient inhibition of cell-associated virus.

These data indicate that the nature of the substituent(s) on the fatty acid chain and the presence of fluorine or azide at the 3′-position are determinants of anti-HIV activity. The lower activities of certain analogs may be due to the slower regeneration of the parent nucleoside or slower cellular uptake. The ability of fatty acyl derivatives of FLT such as 5 and 8 control the cell-to-cell transmission of the virus, whereas FLT is not active at all, which could be due to their higher cellular uptake compared to FLT in the infected cells and enhanced the delivery of FLT in infected cells. Additionally, 5 and 8 also release myristic acid analogs intracellularly that can potentially inhibit the post-translation modification of viral proteins such as protein myristoylation, and eventually block the release of new virus to infect other cells.

#### 2.3.2. Anti-HIV Activities against MDR Isolates

Based on a preliminary screening, conjugates **5** and **8** were evaluated for their ability to inhibit a drug-resistant virus in a peripheral blood mononuclear cell (PBMC) assay. In this assay, FLT, **5**, and **8** were tested against B- and C-wild type and A-17 and 4755–5 multidrug-resistant (MDR) clinical isolates of HIV-1, where compounds were incubated with PBMCs for 6 h and then washed off before the addition of the virus and further culture for 7 days. Results indicated that FLT derivatives **5** (IC_50_ = 1.1 and 2.1 nM) and **8** (IC_50_ = 0.6 and 1.0 nM) moderately improved the overall activity against B- and C-wild type virus, respectively, in comparison to the FLT (IC_50_ = 2.0 and 2.0 nM). More importantly, **5** and **8** were significantly more active against the drug-resistant virus compared to FLT (Table 4). In comparison to FLT (IC_50_ = 26.0 and 107.3 nM), compound **5** (IC_50_ = 4.4 and 39.6 nM) was at least 6- and 2.7-fold more potent against the A-17 and 4755-5 strains of the MDR virus, respectively. However, compound **8** (IC_50_ = 13.6 and 111.5 nM) was only twice as active against the A-17 MDR strain of the virus and was equally active against the 4766-5 MDR strain of the virus. These results indicate that **5** and **8** offer better activity against the wild type as well as drug-resistant virus, where FLT fails as a parent nucleoside.

#### 2.3.3. Cellular Uptake Study

The cellular uptake profile of 5′-*O*-fatty acyl derivatives were investigated in comparison with FLT to support the hypothesis of improved antiviral activity for conjugates such as **5** and **8**. FLT attached to FAM through β-alanine (**23**, shorter linker) was used as a control FLT analog. FLT attached to FAM through 12-aminododecanoic acid (**24**, longer linker) was used as an analog of 3′-fluoro-2′,3′-dideoxy-5′-*O*-(12-azidododecanoyl)thymidine (**5**) and other fatty acid ester conjugates of FLT. 3′-Fluoro-2′,3′-dideoxy-5′-*O*-(12-aminododecanoyl)thymidine (**22**) showed anti-HIV activities comparable to other fatty acyl derivatives of FLT (Table 2). The fluorescein-labeled fatty acyl ester derivative of FLT (**24**) showed a slightly lower anti-HIV activity when compared with the unsubstituted 12-aminododecanoyl derivative (**22**).

**Figure 1:**
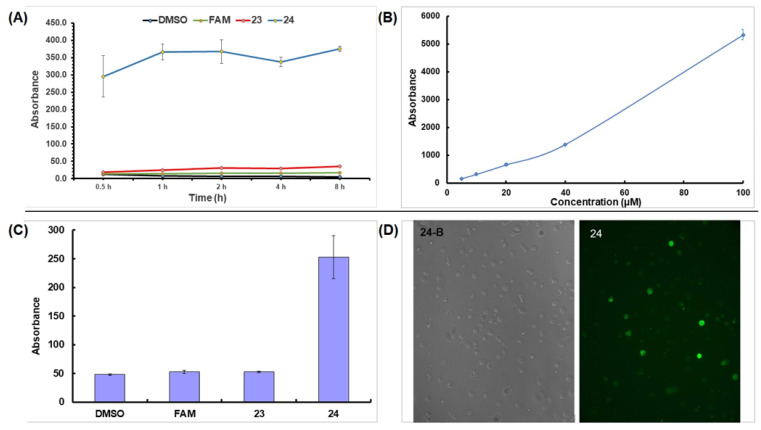
(**A**) Cellular uptake studies for 10 µM of 5(6)-carboxyfluorescein derivatives of FLT (**23** and **24**) along with FAM and DMSO as the controls for the different time intervals. (**B**) Cellular uptake studies for the 5(6)-carboxyfluorescein derivative of FLT (**24**) after 1 h at different concentrations. (**C**) Cellular uptake studies for 10 µM of 5(6)-carboxyfluorescein derivatives of FLT (**23** and **24**) along with FAM and DMSO as controls after treatment with trypsin. (**D**) Real-time fluorescence microscopy in live CCRF-CEM cell line after 1 h incubation of **24** (10 µM).

Human T-lymphoblastoid cells (CCRF-CEM, ATCC No. CCL-119) were used in the study and were grown to 70% confluency in the culture media. The cells were incubated with the fluorescein-substituted conjugates (**23** and **24**) for different time periods (Figure 1A) and in incremental concentrations of **24** (Figure 1B). DMSO and FAM were used as a control for the study. The cells were analyzed by flow cytometry (FACSCalibur: Becton Dickinson) using the FITC channel and CellQuest software. The data presented are based on the mean fluorescence signal for 10,000 cells collected. All of the assays were carried out in triplicate.

In the first assay, the CCRF-CEM cells were incubated with 10 µM of the compounds for different time periods (0.5 h, 1 h, 2 h, 4 h, and 8 h, Figure 1A). Compound **24** exhibited a 10–15-fold higher cellular uptake than that of **23** and FAM alone. The results clearly indicate that the presence of a long-chain enhances the cellular uptake of FLT by increasing the lipophilicity. The continuous incubation of cells with compounds up to 8 h did not show a significant difference in the cellular uptake, suggesting that most of the fatty acyl ester derivative is absorbed in the cells within the first 60 min of incubation.

In the second assay, cells were incubated with different concentrations (5, 10, 20, 40, and 100 µM) of **24** for 1 h (Figure 1B). The data suggest that the cellular uptake was concentration-dependent. In the third assay, we used trypsin wash to confirm that the enhanced uptake of **24** was not due to the absorption on the cell membrane surface. In this assay, cells were incubated with 10 µM of DMSO, FAM, **23**, and **24** for 1 h and then treated with trypsin for 5 min to wash the adsorbed molecules (if any) from the cell membrane. The cellular uptake studies after trypsin treatment showed that the cellular uptake of **24** was still about 5 times higher than those of the control compounds, FAM and **23** (Figure 1C), suggesting that the higher cellular uptake of **24** was not due to artificial absorption to the cell membrane.

Cells incubated with 10 µM of DMSO, FAM, **23** and **24** for 1 h were used for real time fluorescence microscopy using transmitted light microscopy equipped with a differential-interference contrast method and an Achroplan 40× objective. Cells showed no significant fluorescence when incubated with DMSO, FAM, and **23**. On the other hand, cells incubated with 10 µM of **24** showed visible fluorescence (Figure 1D).

Cellular uptake studies using **24** (fluorescent equivalent of **5** or **8**) or **23** (the fluorescent equivalent of FLT) indicated that fatty acyl conjugates were able to rapidly enter the cells in higher concentrations compared to the parent nucleosides. In general, these data indicate that the fatty acyl derivatives of nucleosides have better cellular uptake than their parent nucleosides, and the improved cellular uptake must be the contributing factor in improved antiviral activity.

#### 2.3.4. Cell Viability Study

Cell viability study was performed to analyze the effect of FAM, **23**, and **24** on the live cells. The CCRF-CEM cells were incubated with 10 µM of the compounds and mixed with trypan blue (0.1%) to color the dead cells. The percentage of viability was calculated by using Cellometer Auto T.4 (Nexcelom Bioscience). It was observed that at least 80% of the cells were viable in the presence of the compounds in a 24 h incubation period (Supporting Materials, Figure S1).

#### 2.3.5. Effect of Conjugates on Sperm Viability and Mobility

In this study, several fatty acyl conjugates of nucleosides (**5**–**8**, **10**–**12**, **15**, and **16**) were tested for their spermicidal activities. In a dose–response study to evaluate spermicidal activity, none of the derivatives characterized in this study showed any significant sperm immobilizing or spermicidal activity, even at their maximum concentrations (1 mg/mL). Detailed data analysis is available in the Supplementary Materials (Figure S2).

#### 2.3.6. Effect of Conjugates on Vaginal Cell Viability

**Figure 2:**
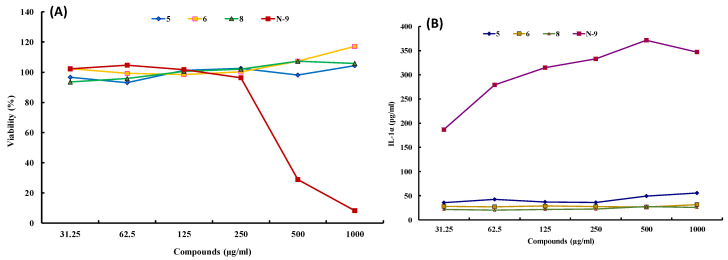
(**A**) Dose–response curves of the vaginal cytotoxicity in VK-2 cells (MTS assay) for **5**, **6**, **8**, and N-9 after 6 h incubations. (**B**) Proinflammatory cytokine (IL-1α) production in the VK-2 cells (ELISA) after a 6 h incubation in the presence of **5**, **6**, **8**, and N-9.

A commercially used spermicidal agent (N-9) and conjugate cytotoxicity was evaluated using immortalized human vaginal cells (VK-2/E6E7). Conjugates **5**, **6**, and **8** showed significantly less cytotoxicity and proinflammatory potential than N-9 (Figure 2), while displaying potent anti-HIV activity. In contrast to N-9 (used as positive control), **5**, **6**, and **8** did not show significant cytotoxic effects during a 6 h incubation at multiple concentrations (ranging from 31–1000 µg/mL) (Figure 2A). The vaginal cell viability decreased after incubating cells with N-9, especially at higher concentrations. Observed cytotoxicity from N-9 could be correlated with the generation of higher levels of cytokines by N-9. It was found that N-9 produced a very high concentration of proinflammatory cytokines such as IL-1α in comparison to the conjugates. Unlike N-9, compounds **5**, **6**, and **8** did not induce the release of IL-1α, a powerful proinflammatory cytokine (Figure 2B). These results indicate that the conjugates had only improved the antiviral activity without compromising the safety profile against human cells (vaginal).

## 3. Materials and Methods

### 3.1. Materials

For the initial work, FLT was synthesized at a 5 g scale according to the previously reported method [33]. More FLT and AZT were purchased from Euro Asia Tran Continental (Bombay, India) for large-scale synthesis of the ester conjugates. 12-Bromododecanoic acid was purchased from the Sigma Aldrich Chemical Co (St. Louis, MO, USA). 5(6)-Carboxyfluorescein (FAM) was purchased from Novabiochem-Millipore (Burlington, MA, USA). All of the other reagents including solvents were purchased from Fisher Scientific (Hampton, NI, USA). Human T-lymphoblastoid cells (CCRF-CEM, ATCC No. CCL-119), the HeLa (human cervical carcinoma: ATCC CCL-2.1) cell line, and human vaginal cells (VK-2/E6E7, ATCC CRL-2616) were purchased from ATCC (Manassas, VA, USA).

The products were purified on a Phenomenex^®^Gemini 10 µm ODS reversed-phase column (2.1 × 25 cm) with a Hitachi HPLC system using a gradient increment in the acetonitrile (0.1%TFA) concentration from 0 to 100% in 45 min at a constant flow rate of 17 mL/min. The purity of the compounds was confirmed by using an analytical Hitachi analytical HPLC system on a C18 column (Grace Allsphere ODS 2–3 µ, 150 × 4.6 mm) using a gradient system (water:acetonitrile 30:70 *v*/*v*) at a constant flow rate of 1 mL/min with a UV detection at 265 nm. The chemical structures of the final products were characterized by nuclear magnetic resonance spectrometry (^1^H NMR and ^13^C NMR) determined on a Bruker NMR spectrometer (400 MHz, Daltonik, Bremen, Germany) and confirmed by a high-resolution PE Applied Biosystems Mariner API time-of-flight electrospray mass spectrometer (Waltham, MA, USA). Chemical shifts are reported in parts per millions (ppm). For cellular uptake studies, cells were analyzed by flow cytometry (FACSCalibur: Becton Dickinson, San Jose, CA, USA) using the FITC channel and CellQuest software 7.5.3 (Becton Dickinson, San Jose, CA, USA). Cell-viability studies were conducted using Cellometer Auto T.4 (Nexcelom Biosciences, Lawrence, MA, USA). The real-time microscopy in a live CCRF-CEM cell line with or with compounds was imaged using a ZEISS (Jena, Germany) Axioplan 2 light microscope equipped with transmitted light microscopy with a differential–interference contrast method and an Achroplan 40× objective.

### 3.2. Chemistry

General procedure for the synthesis of 5′-*O*-(fatty ether) derivatives of FLT and AZT: 3′-fluoro-2′,3′-dideoxy-5′-*O*-(tetradecanyl)thymidine (**3**) and 3′-azido-2′,3′-dideoxy-5′-*O*-(tetradecanyl)thymidine (**4**). Ether derivatives of FLT and AZT were synthesized by using Mitsunobu reaction. AZT or FLT (100 mg, 0.4 mmol), tetradecanol (0.8 mmol), and TPP (210 mg, 0.8 mmol) were dissolved in DMF (10 mL). To the reaction mixture was added DIAD (100 mg, 0.5 mmol)). The mixture was stirred for 5 h at room temperature. The solvent was removed in vacuo. The residue was purified by reversed-phase HPLC using a C_18_ column and water/acetonitrile as the solvents as described above. The chemical structures of compounds were confirmed by ^1^H NMR and ^13^C NMR (Figures S3–S6).

**3′-Fluoro-2′,3′-dideoxy-5′-*O*-(tetradecanyl)thymidine (3).** White powder; yield (80 mg, 50%); ^1^H NMR (400 MHz, CDCl_3_, δ ppm): 7.52 (s, 1H, H-6), 6.19 (dd, *J* = 9.0 and 5.6 Hz, 1H, H-1′), 5.18 (dd, *J* = 54.0 and 4.6 Hz, 1H, H-3′), 4.18 (d, *J* = 27.5 Hz, 1H, H-4′), 3.65–3.86 (m, 4H, *CH_2_*O, H-5′ and H-5″), 2.34–2.52 (m, 1H, H-2′), 2.10–2.34 (m, 1H, H-2″), 1.77 (s, 3H, 5-C*H_3_*), 1.37–1.55 (m, 2H, C*H_2_*CH_2_O), 1.05–1.24 (br s, 20 H, methylene envelope), 0.74 (s, 3H, C*H_3_*); ^13^C NMR (CDCl_3_, 100 MHz, δ ppm): 163.35 (C-4 *C*=O), 150.65 (C-2 *C*=O), 134.57 (C-6), 109.98 (C-5), 94.37 (d, *J* = 176.8 Hz, C-3′), 86.75 (C-1′), 85.20 (d, *J* = 24.2 Hz, C-4′), 61.95 (d, *J* = 11.0 Hz, C-5′), 41.28 (*CH_2_*-O), 38.15 (d, *J* = 20.9 Hz, C-2′), 32.45, 31.65, 29.38, 29.28, 29.22, 29.08, 27.31, 26.72, 25.54, 22.41 (methylene carbons), 13.81 (*C*H_3_), 12.94 (5-*C*H_3_). HR-MS (ESI-TOF) (m/z): C_24_H_41_FN_2_O_4_: calcd. 440.305; found 441.1052 [M + H]^+^.

**3′-Azido-2′,3′-dideoxy-5′-*O*-(tetradecanyl)thymidine (4).** White powder; yield (90 mg, 50%. ^1^H NMR (400 MHz, CDCl_3_, δ ppm): 7.35 (s, 1H, H-6), 6.02 (t, *J* = 6.6 Hz, 1H, H-1′), 4.35 (dd, *J* = 5.1, 11.5 Hz, 1H, H-3′), 3.88–3.96 (m, 2H, H-5′ and H-5″), 3.85 (t, *J* = 7.3 Hz, 1H, *CH_2_*O), 3.76 (d, *J* = 10.6 Hz, 1H, H-4′), 2.40–2.54 (m, 1H, H-2′), 2.28–2.40 (m, 1H, H-2″), 1.86 (s, 3H, 5-C*H_3_*), 1.53 (t, *J* = 6.3 Hz, 2H, C*H_2_*CH_2_O), 1.12–1.30 (br s, 20H, methylene envelope), 0.82 (t, *J* = 6.6 Hz, 3H, C*H_3_*); ^13^C NMR (CDCl_3_, 100 MHz, δ ppm): 163.28 (C-4 *C*=O), 150.72 (C-2 *C*=O), 134.58 (C-6), 110.29 (C-5), 87.23 (C-1′), 84.47 (C-4′), 61.89 (C-5′), 59.90 (C-3′), 41.46 (*CH_2_*O), 37.35 (C-2′), 31.85, 29.58, 29.53, 29.48, 29.28, 29.24, 27.51, 26.93, 22.62, (methylene carbons), 14.05 (*C*H_3_), 13.22 (5-*C*H_3_). HR-MS (ESI-TOF) (m/z): C_24_H_41_N_5_O_4_: calcd. 463.3159; found 464.1528 [M + H]^+^.

**5′-*O*-(Fatty acyl) ester derivatives of FLT and AZT**. First, several 5′-*O*-(fatty acyl) ester derivatives of FLT and AZT were synthesized at a scale of 100 mg according to the previously reported procedure [9,10] by the reaction of FLT or AZT and in situ generated fatty acyl chloride derivatives in the presence of 4-dimethylaminopyridine (DMAP). In the next step, three FLT esters 3′-fluoro-2′,3′-dideoxy-5′-*O-*(12-azidododecanoyl)thymidine (**5**), [5′-*O-*(myristoyl)-3′-fluoro-2′,3′-dideoxythymidine (**6**), and 3′-fluoro-2′,3′-dideoxy-5′-*O-*(12-thioethyldodecanoyl)thymidine (**8**)] were synthesized at a larger scale of 5 g and 25 g as we previously reported [9]. Similarly, 5′-*O*-(fatty acyl) ester derivatives of AZT or FLT (**7**, **9**, **10**, **11**, **12**, **13**, **16**, **17**, **18**) were synthesized as described previously [9,10].

In general, a reaction mixture consisting of the appropriate fatty acid (1.3 mmol), oxalyl chloride (0.25 g, 1.95 mmol), and anhydrous benzene (18 mL) was stirred at room temperature (25 °C) for 1 h. The obtained yellow solution was evaporated to dryness under reduced pressure. The residual oil was dissolved in benzene (18 mL), and the solution was added dropwise to an ice-cold, stirred solution consisting of the AZT (0.34 g, 1.3 mmol) or the FLT (0.32 g, 1.3 mmol), DMAP (0.23 g, 1.9 mmol), and anhydrous benzene (18 mL) under anhydrous conditions. The solution was stirred in an ice bath for 1 h and then refluxed in an oil bath for about 3 h. The mixture was cooled and diluted with benzene (100 mL). The organic solution was washed with saturated aqueous sodium carbonate (2 × 25 mL) and then with water (2 × 25 mL). The organic layer was dried over anhydrous sodium sulfate and was evaporated to dryness. The residue consisting of one major product was purified by silica gel chromatography using chloroform as an eluent to yield the product. The procedure was used for the synthesis of most of the ester analogs unless noted otherwise.

**(±)-3′-Fluoro-2′,3′-dideoxy-5′-*O*-(2-methoxyteradecanoyl)thymidine (13).** Oil; yield (100 mg, 90%); ^1^H NMR (400 MHz, CDCl_3_, δ ppm): 8.50 (s, 1H, N*H*), 7.31 (s, 1H, H-6), 6.40 (dd, *J* = 9.0 and 5.6 Hz, 1H, H-1′), 5.08–5.26 (dd, *J* = 52.5 and 5.5 Hz, 1H, H-3′), 4.46 (dt, *J* = 25.6 and 4.0 Hz, 1H, H-4′), 4.55 (dd, *J* = 12.5 and 4.1 Hz, 1H, H-5′), 4.25 (dd, *J* = 12.5 and 4.1 Hz, 1H, H-5″), 3.38 (t, *J* = 7.6 Hz, 1H, C*H*CO), 1.95 (s, 3H, 5-CH_3_), 2.55–2.72 (m, 1H, H-2″), 2.15–2.25 (m, 1H, H-2′), 1.70–1.80 (m, 2H, C*H*_2_CH(OCH_3_)), 1.32–1.42 (m, 2H, C*H*_2_CH_2_CH(OCH_3_)), 1.20–1.30 (br m, 18H, methylene protons), 0.87 (t, *J* = 7.6 Hz 3H, CH_3_). HR-MS (ESI-TOF) (m/z): C_25_H_41_FN_2_O_6_, calcd, 484.2949; found, [M + Na]^+^_._

**(±)-3′-Azido-2′,3′-dideoxy-5′-*O*-(2-methoxyteradecanoyl)thymidine (14).** Oil; yield (100 mg, 90%); ^1^H NMR (400 MHz, CDCl_3_, δ ppm): 9.00 (s, 1H, N-*H*), 7.28 (s, 1H, H-6), 6.17 (t, *J* = 6.2 Hz, 1H, H-1′), 4.50 (dd, *J* = 12.2 and 3.7 Hz, 1H, H-5*^′^*), 4.35 (dd, *J* = 12.2 and 3.7 Hz, 1H, H-5*^″^*), 4.20 (ddd, *J =* 7.6, 6.2, and 5.0 Hz, 1H, H-3*^′^*), 4.10 (ddd, *=* 5.0, 3.7, and 3.0 Hz, 1H, H-4*^′^*), 3.38 (t, *J* = 7.6 Hz, 1H, C*H*CO), 3.39 (s, 3H, OC*H*_3_), 2.50 (ddd, *J* = 13.9, 6.2, and 6.2 Hz, 1H, H-2″), 2.37 (ddd, *J* = 13.9, 7.6 and 6.2 Hz, H-2*^″^*), 1.95 (s, 3H, 5-C*H*_3_), 1.70–1.80 (m, 2H, C*H*_2_CH(OCH_3_)), 1.32–1.42 (m, 2H, C*H*_2_CH_2_CH(OCH_3_)), 1.22–1.31 (br m, 18H, methylene protons), 0.89 (t, *J* = 7.6 Hz, 3H, CH_3_); HR-MS (ESI-TOF) (m/z): C_25_H_41_N_5_O_6_, calcd, 507.3057; found, 530.6788 [M + Na]^+^.

General procedure for the synthesis of 3′-fluoro-2′,3′-dideoxy-5′-*O*-(3-aminopropanoyl)thymidine (**21**) and 3′-fluoro-2′,3′-dideoxy-5′-*O*-(12-aminododecanoyl)thymidine (**22**). FLT (0.60 mmol, 150 mg), the appropriate Fmoc-amino acid (1.2 mmol), and 2-(1H-benzotriazole-1-yl)-1,1,3,3-tetramethyluronium hexafluorophosphate (HBTU, 500 mg, 1.3 mmol) were dissolved in dry DMF (10 mL) and dry *N*-methylmorpholine (2 mL). The solution was stirred overnight at room temperature. The reaction mixture was concentrated and dried under reduced pressure to afford the crude 5′-*O*-Fmoc-amino acid derivatives of FLT, **19**, and **20**. The crude intermediates were dissolved as such in THF (10 mL). To the reaction mixture was added piperidine (6 μL, 0.06 mmol), 1-octanethiol (10 mM solution in THF, 6 mmol, 0.6 mL). The reaction mixture was allowed to stir for 1 h at room temperature. The reaction solution was concentrated at reduced pressure. The residue was purified with reversed-phase HPLC using a C_18_ column and using a gradient system with water/acetonitrile as the solvents, as described above. The chemical structures of the compounds were confirmed by ^1^H NMR and ^13^C NMR as shown for compound **22** in Figures S7 and S8.

**3′-Fluoro-2′,3′-dideoxy-5′-*O*-(3-aminopropanoyl)thymidine (21).** White powder; yield (100 mg, 55%). HR-MS (ESI-TOF) (m/z): C_13_H_18_FN_3_O_5_: calcd. 315.1230; found 316.3369 [M + H]^+^, 631.4397 [2M + H]^+^.

**3′-Fluoro-2′,3′-dideoxy-5′-*O*-(12-aminododecanoyl)thymidine (22).** White powder; yield (150 mg, 57%). ^1^H NMR (400 MHz, CD_3_OD, δ ppm): 11.38 (s, 1H, N*H*), 7.81–7.98 (br s, 2H, N*H_2_*), 7.45 (s, 1H, H-6), 6.18 (t, *J* = 7.9 Hz, H-1′), 5.30 (dd, *J* = 53.2 and 4.2 Hz, 1H, H-3′), 4.15–4.36 (m, 3H, H-5′, H-5″, H-4′), 3.15 (t, *J* = 8.2 Hz, 2H, *CH_2_*NH), 2.68–2.82 (m, 2H, *CH_2_*CO), 2.21–2.52 (m, 4H, *CH_2_*CH_2_NH, H-2′, H-2″), 1.76 (s, 3H, 5-C*H_3_*), 1.05–1.24 (br m, 16H, methylene envelope); ^13^C NMR (DMSO-d_6_, 100 MHz, δ ppm): 173.08 (COO), 164.07 (C-4 *C*=O), 150.87 (C-2 *C*=O), 136.08 (C-6), 110.03 (C-5), 94.38 (d, *J* = 175.5 Hz, C-3′), 84.66 (C-1′), 82.03 (d, *J* = 25.8 Hz, C-4′), 63.49 (d, *J* = 10.6 Hz, C-5′), 55.32, 51.54 (*C*H_2_NH), 36.33 (d, *J* = 20.6 Hz, C-2′), 33.73 (*C*H_2_COO), 29.30, 29.25, 29.12, 28.96, 28.91, 28.84, 27.41, 26.23, 24.79, 23.51 (methylene carbons), 13.86 (5-*C*H_3_). HR-MS (ESI-TOF) (m/z): C_22_H_36_FN_3_O_5_: calcd. 441.5367; found 442.1974 [M + H]^+^, 883.5248 [2M + H]^+^.

**General procedure for the synthesis of 5′-*O*-(5(6)-carboxyfluorescein) derivatives of FLT (23 and 24).** FLT was attached to 5(6)-carboxyfluorescein (FAM) through β-alanine (**21**) or 12-aminododecanoic acid (**22**) as linkers. A mixture of 5(6)-carboxyfluorescein (430 mg, 1.15 mmol), the corresponding FLT-amino acid (1.3 and 1.4, 0.29 mmoL) and HBTU (440 mg, 1.15 mmol) was dissolved in dry DMF (10 mL) and diisopropylethylamine (DIPEA, 2 mL, 15 mmol) and stirred overnight at room temperature. The reaction mixture was concentrated and dried under vacuum. The final compounds were purified with reversed-phase HPLC using a C_18_ column and water/acetonitrile as the solvents, as described above. The chemical structures of the compounds were confirmed by ^1^H NMR and ^13^C NMR (Figures S9–S12).

**3′-Fluoro-2′,3′-dideoxy-5′-*O*-(3-(N(5(6)-carboxyfluorescein)aminopropanoyl)thymidine (23).** Yield (25 mg, 15%). ^1^H NMR (400 MHz, CD_3_OD, δ ppm): 8.48 (s, 0.5H, FAM-Ar-H, 5 or 6 isomer), 8.19 (d, *J* = 8.2 Hz, 0.5H, FAM-Ar-H, 5 or 6 isomer), 8.04, 8.13 (two dd, *J* = 1.6 and 8.0 Hz, 1H, FAM-Ar-H), 7.58 (s, 0.5H, FAM-Ar-H, 5 or 6 isomer), 7.20–7.42 (m, 1H, H-6, and 0.5 H FAM-Ar-H, 5 or 6 isomer), 6.96–7.05 (m, 4H, FAM-Ar-H), 6.84 (d, *J* = 8.9 Hz, 2H, FAM-Ar-H), 6.11 and 6.17 (two dd, *J* = 5.6 and 8.9, 1H, H-1′), 5.16 and 5.23 (two dd, *J* = 53.3 and 5.0 Hz, 1H, H-3′), 4.20–4.40 (m, 3H, H-5′ and H-5″, H-4′), 3.53 and 3.64 (two t, *J* = 6.5 Hz, 2H, *CH_2_*NH), 2.10–2.74 (m, 4H, H-2′, H-2″, *CH_2_*CO), 1.72 and 1.78 (two s, 3H, 5-C*H_3_*); ^13^C NMR (CD_3_CN, 100 MHz, δ ppm): 172.63 (COO), 172.47 (NHCO), 168.06, 165.63, 156.25 (FAM-Ar-*C*), 160.49 (C-4 *C*=O), 151.20, 151.14 (C-2 *C*=O), 139.64, 133.27, 131.43, 129.24, 129.60, 127.37, 127.82, 126.07 (FAM-Ar-*C*), 136.46, 136.52 (C-6), 116.84 (FAM-Ar-*C*), 113.47, 113.58 (C-5), 102.83 (FAM-Ar-*C*), 94.31, 94.19 (two d, *J* = 176.5 Hz, C-3′), 85.53, 85.57 (C-1′), 82.57, 82.48 (two d, *J* = 26.8 Hz, C-4′), 64.13, 64.00 (two d, *J* = 11.2 Hz, C-5′), 55.11, 49.09 (*C*H_2_NH), 36.07, 35.97 (C-2′), 33.76, 33.89 (*C*H_2_COO), 11.95, 12.01 (5-*C*H_3_). HR-MS (ESI-TOF) (m/z): C_34_H_28_FN_3_O_11_: calcd. 673.1708; found 674.5083 [M + H]^+^, 1348.6951 [2M + H]^+^.

**3′-Fluoro-2′,3′-dideoxy-5′-*O*-(12-(N(5(6)-carboxyfluorescein)aminododecanoyl)thymidine (24).** Yield (30 mg, 13%); ^1^H NMR (400 MHz, CD_3_OD, δ ppm): 8.45 (s, 0.5H, FAM-Ar-H, 5 or 6 isomer), 8.17 (dd, *J* = 1.5 and 8.0 Hz, 0.5H, FAM-Ar-H, 5, or 6 isomer), 8.10 (s, 1H, FAM-Ar-H), 7.64 (s, 0.5H, FAM-Ar-H, 5, or 6 isomer), 7.43 (s, 1H, H-6), 7.30 (d, *J* = 5.1 Hz, 0.5H, FAM-Ar-H, 5 or 6 isomer), 6.76 (s, 1H, FAM-Ar-H), 6.70 (dd, *J* = 3.2 and 8.7 Hz, 3H, FAM-Ar-H), 6.61 (dd, *J* = 2.0 and 8.7 Hz, 2H, FAM-Ar-H), 6.16–6.26 (m, 1H, H-1′), 5.20 (dd, *J* = 53.4 and 4.9 Hz, 1H, H-3′), 4.30–4.45 (m, 2H, H-5′ and H-5″), 4.19 (d, *J* = 5.5 and 13.6 Hz, 1H, H-4′), 3.38 (t, *J* = 7.0 Hz, 2H, *CH_2_*NH), 2.44–2.63 (m, 1H, H-2′), 2.15–2.38 (m, 3H, *CH_2_*CO, H-2″), 1.83 (s, 3H, 5-C*H_3_*), 1.47–1.66 (m, 4H, *CH_2_*CH_2_COO, *CH_2_*CH_2_-NH), 1.05–1.24 (br m, 16H, methylene envelope); ^13^C NMR (CD_3_OD, 100 MHz, δ ppm): 174.85 (COO), 170.11 (NHCO), 168.34, 166.35, 155.35 (FAM-Ar-*C*), 163.45 (C-4 *C*=O), 152.29 (C-2 *C*=O), 142.15, 138.28, 137.40, 131.01, 129.46, 126.86, 126.08, (FAM-Ar-*C*), 135.12 (C-6), 115.14 (FAM-Ar-*C*), 112.02 (C-5), 103.72 (FAM-Ar-*C*), 95.32 (d, *J* = 177.6 Hz, C-3′), 86.92 (C-1′), 84.15 (d, *J* = 26.3 Hz, C-4′), 64.66 (d, *J* = 11.7 Hz, C-5′), 54.98, 41.40 (*C*H_2_NH), 38.83 (d, *J* = 21.0 Hz, C-2′), 35.02 (*C*H_2_COO), 30.77, 30.74, 30.68, 30.59, 30.54, 30.45, 30.41, 30.34, 30.26, 30.21, 28.18, 26.10 (methylene carbons), 12.81 (5-*C*H_3_). HR-MS (ESI-TOF) (m/z): C_43_H_46_FN_3_O_11_: calcd. 799.3116; found 800.4325 [M + H]^+^.

### 3.3. Physicochemical Properties (pKa, Log D., Solubility)

The physicochemical properties including pKa (9.67 ± 0.02), Log D (5.04, pH = 7.4), and solubility were determined for **5** as a model compound.

#### 3.3.1. pKa

The pKa for **5** was determined using dip-probe absorption spectroscopy (D-PAS) [34]. The sample was initially titrated in a fast titration between pH 1.8 and pH 12.1 at concentrations of 33–49 µM under aqueous conditions. Precipitation of the sample from the solution was observed below approximately pH 11. The sample was subsequently titrated under methanol–water co-solvent conditions in two triple titrations from pH 12.2 to pH 3.7 at concentrations of 31–49 µM. The methanol ratio varied from to 25.5–50.8%. No precipitation of the sample from the solution was observed under this condition. The pKa was determined from the spectroscopic data by the Yasuda–Shedlovsky extrapolation of the individual obtained results. Compound **5** was found to have an aqueous pKa value of 9.67 ± 0.02 as determined by the spectroscopic method under methanol–water co-solvent conditions.

#### 3.3.2. Log P and Log D

The Log P of **5** was initially investigated by the pH-metric (potentiometric) method. The sample was titrated in three triple titrations from pH 2.5 to pH 11.9 at concentrations of 0.7–1.1 mM in various ratios of octanol/water. The results indicated high sample lipophilicity, although the Log P could not be determined due to the apparent pKa in octanol shifting out of the measurable range. Therefore, the Log D of **5** at pH 7.4 was measured as 5.04 using liquid–liquid distribution chromatography. The Profiler LDA is an isocratic chromatography system that uses an octanol-coated column with octanol saturated mobile phases adjusted to pH 7.4. A set of standard compounds with well-known Log D octanol values were run through the column before the samples, and the generated retention times were used as a calibration curve to relate the retention times generated for the sample compounds to Log D. Detection was carried out by using a UV diode array. A multi-wavelength peak location system was used to hone in on the largest peak present in the chromatogram. A Log D value of 5.04 was obtained, which should correspond to the neutral Log P, in consideration of the sample pKa.

#### 3.3.3. Solubility

Solubility for **5** was investigated by the shake-flask methodology with UV-spectroscopic sample detection. To produce a saturated solution of **5**, approximately 1 mg of solid material was added to 10 mL of an aqueous solution, which was adjusted to pH 2.3 with 0.5 M HCl. The sample was then sonicated in an ultrasonic bath for several hours (at room temperature) before being left to equilibrate for a period of approximately 3 days. The supernatant was then filtered under vacuum through a 0.2 µm PVDF filter plate, and the UV absorption spectrum of the sample was measured (after adjusting the pH of the solution to 11.8 with 0.5 M KOH). Molar absorption coefficients of **5** were obtained at pH 11.8 for 25 µM, 50 µM, and 100 µM solutions of **5**, in order to quantify the concentration of the sample in the saturated supernatant. The UV-absorption signal (0.0039) of the supernatant at the absorption maximum of **5** (264 nm, *ε* = 7650 dm^3^cm^−1^Mol^−1^) was close to the detection limit of the apparatus, and the solubility value was determined as an upper limit of <510 nM. Conjugate **5** was completely soluble in ethanol (>30 mg/mL) and the mixture of water/methanol (60:40). However, **5** was less soluble in DMSO (~4.1 mg/mL).

### 3.4. Anti-HIV Assays

Anti-HIV activities of the compounds were evaluated according to the previously reported procedure [35]. In summary, the HeLa (Human cervical carcinoma: ATCC CCL-2.1) cell line was used to measure the inactivation of both the cell-free virus preparations and virus-infected cell preparations. The cells were plated in culture plates 24 h prior to each experiment. Cell-free viral preparations of HIV-1 strains IIIB (lymphocytotropic strain) and BaL (monocytotropic strain) were used for the cell-free assay. For the cell-associated assay, the SulT1 cells were infected with IIIB virus 5 days prior to the experiment. Cell-free virus and virus-infected cells were mixed with different compounds and diluted to make different concentrations. The mixture was further diluted with the buffer and added to the HeLa cells. The cells were incubated at 37 °C for 48 h, stained for β-galactosidase expression, and compared with the β-galactosidase expression from the β-gal-positive cells in the absence of any microbicidal compound to obtain the IC_50_ values.

For the assessment of compounds against the wild type (WT; R5; clones = 94US3393IN [B subtype] and 98USMSC5016 [C subtype]) and drug-resistant (clones = 4755−5 and A17) HIV-1 clinical isolates, PHA-P stimulated cells from at least two normal donors were pooled, diluted in fresh media, and plated in the interior wells of a 96-well round-bottom microplate. Pooling PBMCs from more than one donor was used to minimize the variability observed between the individual donors, which results from the quantitative and qualitative differences in HIV infection and overall response to the PHA and IL-2 of primary lymphocyte populations. Each plate contained virus/cell control wells (cells + virus), experimental wells (drug + cells + virus), and compound control wells (drug + media, no cells, necessary for MTS monitoring of cytotoxicity). Test drug dilutions were prepared in microtiter tubes, and each concentration was placed in appropriate wells. Following the addition of the drug dilutions to the PBMCs, a predetermined dilution of virus stock was then placed in each test well (final MOI ≅ 0.1). Because HIV-1 is not cytopathic to PBMCs, the same assay plate can be used for both the antiviral efficacy and cytotoxicity measurements. Compounds were incubated with virus and cells in a 96-well format for 6 h. The cells were then washed by removing 75% of the medium (150 μL) and replacing it with 150 μL of fresh (no drug) medium. The plates were then centrifuged (∼200 g) for 10 min, after which 150 μL of the medium was removed, and an additional 150 μL of fresh medium was added to each well and further incubated for 6 days or until peak reverse transcriptase (RT) activity was detected. A microtiter plate-based RT reaction was utilized [36]. Incorporated radioactivity (counts per minute, CPM) was quantified using standard liquid scintillation techniques. Compound IC_50_ (50%, inhibition of virus replication) was calculated using statistical software and regression analysis. A cellular uptake study was conducted for fluorescence conjugates **23** and **24** using a previously published procedure with minor modifications [14,15,16]. All of the experiments were conducted in triplicate.

### 3.5. Cell Viability Study

Human T lymphoblastoid cells (CCRF-CEM, ATCC No. CCL-119) were grown to 70% confluency in a culture medium. CCRF-CEM cells were incubated with 10 µM of the compounds and mixed with trypan blue (0.1%) to color the dead cells. The percentage of viability was calculated by using a Cellometer Auto T.4 (Nexcelom Bioscience). The cell viability against the vaginal cells (VK-2/E6E7) was conducted according to the previously reported procedure [37]. The effect of conjugates on the sperm viability and mobility was evaluated as described previously [38].

## 4. Conclusions

Several bifunctional 5′-*O*-fatty acyl derivatives of FLT were designed and synthesized as prodrugs of FLT, a nucleoside reverse transcriptase inhibitor, and their biological activities were evaluated as anti-HIV agents and microbicides. Among all the conjugates, **5**, **6**, and **8** were found to have the most active anti-HIV activity profile. Compared to AZT and FLT, conjugates **5**, **6**, and **8** were active against both the cell-free virus (lymphocytotropic and monocytotropic strains) as well as the cell-associated virus. Conjugates **5**, **6**, and **8** exhibited >4 and >30 times better antiviral index than FLT and AZT, respectively. The increased inhibition by **5**, **6**, and **8** may be due to their increased rate of uptake, resulting in increased intracellular level of active nucleoside achieved by the conjugate. Hydrolysis of the prodrug to the parent analogs was critical for the generation of anti-HIV activity. Presumably, the intracellular hydrolysis yielded two antiviral agents, FLT and fatty acid analog, with different enzyme targets such as RT and NMT. Conjugates **5** and **8** were active against both the R5 and MDR strains. These conjugates have the potential to be used as topical microbicides to prevent HIV infection during sexual activity. These data provide insights into the more rational design of additional potent and safe anti-HIV microbicides using the FLT or other anti-HIV nucleosides as the parent molecule. When taken together, the results will have significant implications for the design of more potent and innovative anti-HIV agents.

## Figures and Tables

**Table 1:**
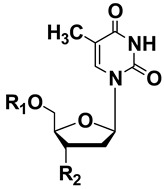
Chemical structures of the 5′-*O*-fatty ether and fatty acyl derivatives of AZT and FLT.

Compd.	R_1_	R_2_	Compd.	R_1_	R_2_
FLT (**1**)	H	F	**12**	Br(CH_2_)_11_CO	N_3_
AZT (**2**)	H	N_3_	**13**	CH_3_(CH_2_)_11_CH(OMe)CO	F
**3**	CH_3_(CH_2_)_13_O	F	**14**	CH_3_(CH_2_)_11_CH(OMe)CO	N_3_
**4**	CH_3_(CH_2_)_13_O	N_3_	**15**	CH_3_(CH_2_)_4_S(CH_2_)_7_CO	F
**5**	N_3_(CH_2_)_11_CO	F	**16**	CH_3_(CH_2_)_4_S(CH_2_)_7_CO	N_3_
**6**	CH_3_(CH_2_)_12_CO	F	**17**	CH_3_(CH_2_)_9_O(CH_2_)_2_CO	N_3_
**7**	CH_3_(CH_2_)_12_CO	N_3_	**18**	CH_3_(CH_2_)_4_O(CH_2_)_7_CO	N_3_
**8**	CH_3_CH_2_S(CH_2_)_11_CO	F	**21**	NH_2_(CH_2_)_2_CO	F
**9**	CH_3_CH_2_S(CH_2_)_11_CO	N_3_	**22**	NH_2_(CH_2_)_11_CO	F
**10**	CH_3_(CH_2_)_13_CO	N_3_	**23**	FAM-NH_2_(CH_2_)_2_CO	F
**11**	Br(CH_2_)_11_CO	F	**24**	FAM-NH_2_(CH_2_)_11_CO	F

**Table 2:** Anti-HIV activities of the fatty acyl ester derivatives of AZT and FLT.

Compd.	Cytotoxicity ^a^ EC_50_^b^ (μM)	(X4) ^c^ EC_50_ (μM)	(R5) ^d^ EC_50_ (μM)	(CA) ^e^ EC_50_ (μM)
FLT, **1**	>410	0.8	0.4	>410
AZT, **2**	>375	10.9	14.2	>375
**3**	179.0	180	176	>227
**4**	205.0	125	27.6	>216
**5**	1598.0	0.9	0.4	12.6
**6**	606.0	0.7	1.1	6.4
**7**	>629	3.1	5.0	>629
**8**	>2000	1.0	<0.2	2.3
**9**	>101	7.7	5.2	90.7
**10**	>611	17.9	4.5	>611
**11**	>198	1.8	<0.2	>198
**12**	>190	14.8	4.6	>190
**13**	>206	1.0	0.2	>206
**14**	>197	9.7	6.7	>197
**15**	>64	11.4	4.4	>64
**16**	>202	9.3	12.9	>202
**17**	>209	6.7	2.1	>209
**18**	>209	4.8	6.1	>209
**22**	>226	3.4	1.6	>227
**24**	>125	6.6	3.6	>123
**C-2** ^f^	>1000	1.6	85.9	5.1
DMSO ^g^	>1000	>1000	>1000	>1000

^a^ Cytotoxicity assay (MTS); ^b^ 50% Effective concentration; ^c^ Single-round infection assay (lymphocytotropic strain); ^d^ Single-round infection assay (monocytotropic strain); ^e^ Cell-associated transmission assay (IIIB); ^f^ Assay control; ^g^ Solvent control. The data represents the average of triplicate experiments.

**Table 3:** Comparison of the anti-HIV activities of fatty acyl derivatives of AZT and FLT with the corresponding physical mixtures of AZT or FLT + fatty acids.

Chemical Name	Cytotoxicity ^a^ EC_50_^b^ (μM)	(X4) ^c^ EC_50_ (μM)	(R5) ^d^ EC_50_ (μM)	(CA) ^e^ EC_50_ (μM)
FLT (**1**)	>410	0.8	0.4	>410
AZT (**2**)	>374	10.9	14.2	>374
C_13_H_27_-COO-FLT (**6**)	606.0	0.7	1.1	6.4
C_13_H_27_-COOH + FLT (50:50) (**25**)	>106	<0.2	0.8	33.0
C_13_H_27_-COO-AZT (**7**)	>629	3.1	5.0	>629
C_13_H_27_-COOH + AZT (50:50) (**26**)	>202	1.4	46.2	>202
Br-C_11_H_22_-COO-AZT (**12**)	>190	14.8	4.6	>190
Br-C_11_H_22_-COOH + AZT (50:50) (**27**)	>183	34.8	8.8	>183

^a^ Cytotoxicity assay; ^b^ 50% Effective concentration; ^c^ Viral entry inhibition assay (lymphocytotropic strain); ^d^ Viral entry inhibition assay (monocytotropic strain); ^e^ Cell-to-cell transmission assay (IIIB). The data represents the average of triplicate experiments.

**Table 4:** Anti-HIV evaluation against wild type clinical isolates and resistant virus ^a^.

Compd.	Virus	Clade/Resistance	IC_50_^b^ (nM)	IC_90_^c^ (nM)
FLT	94US3393IN	B-Wild Type	2.0	20.4
	98USMSC5016	C-Wild Type	2.0	12.3
	A-17 MDR	B-NNRTI	26.0	87.2
	4755-5 MDR	B-MDR	107.3	1065.0
**5**	94US3393IN	B-Wild Type	1.1	6.4
	98USMSC5016	C-Wild Type	2.1	8.6
	A-17 MDR	B-NNRTI	4.4	14.1
	4755-5 MDR	B-MDR	39.6	507.2
**8**	94US3393IN	B-Wild Type	0.6	10.3
	98USMSC5016	C-Wild Type	1.0	8.2
	A-17 MDR	B-NNRTI	13.6	45.7
	4755-5 MDR	B-MDR	111.5	662.2

^a^ Multi-round infection assay using HIV-1 clinical isolates [wild type R5 clones = 94US3393IN (B subtype) and 98USMSC5016 (C subtype) and multi-drug resistant clones = 4755−5 and A17] HIV-1 clinical isolates. ^b^ IC_50_ = The minimum drug concentration that inhibits HIV-induced cytopathic effect by 50%, calculated by using a regression analysis program for semilog curve fitting; ^c^ IC_90_ = The minimum drug concentration that inhibits HIV-induced cytopathic effect by 90%, calculated by using a regression analysis program for semilog curve fitting). The data represent the average of triplicate experiments.

## Data Availability

Not applicable.

## Supplementary Materials

The following supporting information can be downloaded at: https://www.mdpi.com/article/10.3390/molecules27103352/s1. Figure S1. Cell viability assay after 3 h and 24 h of incubation of **23** and **24** with CCRF-CEM cells; Figure S2. In vitro assays for the spermicidal activity of fatty acyl conjugates of FLT and AZT in comparison to N-9; Figure S3. ^1^H NMR of **3**; Figure S4. ^13^C NMR of **3**; Figure S5. ^1^H NMR of **4**; Figure S6. ^13^C NMR of **4**; Figure S7**.** ^1^H NMR of **22**; Figure S8. ^13^C NMR of **22**; Figure S9. ^1^H NMR of **23**; Figure S10. ^13^C NMR of **23**; Figure S11. ^1^H NMR of **24**; Figure S12. ^13^C NMR of **24**.

## Sample Availability

Compound **8** is available from K. Parang.
