# Supplementary material for: Synthesis and Biological Evaluation of 5′-O-Fatty Acyl Ester Derivatives of 3′-Fluoro-2′,3′-dideoxythymidine as Potential Anti-HIV Microbicides
Source: Molecules. 2022 May 23;27(10):3352. doi: 10.3390/molecules27103352 (PMC9143043; doi:10.3390/molecules27103352)
Supplement: Supplementary file 1 [file molecules-27-03352-s001.zip › molecules-1717314-supplementary.pdf]

SUPPORTING INFORMATION

Synthesis and Biological Evaluation of 5′-*O*-Fatty Acyl Ester Derivatives of 3′-Fluoro-2′,3′-Dideoxythymidine as Potential Anti-HIV Microbicides

Hitesh K. Agarwal,[a,b,*] *Bhupender Singh Chhikara,*[b] *Guofeng Ye,*[b] *Sitaram Bhavaraju,*[b] *Ajay Dixit,*[b,c] *Anil Kumar,*[b] *Gustavo F. Doncel*[d,*] *Keykavous Parang*[b,e,*]

[a]Department of Pharmaceutical Sciences, School of Pharmacy, South University, 709 Mall Boulevard, Savannah, GA 31406

[b]Department of Biomedical and Pharmaceutical Sciences, School of Pharmacy, University of Rhode Island, 02881, USA

[c]ITC Life Science & Technology Center, #3, 1st Main, Peenya Industrial Area, 1st Phase, Bangalore, 560058, INDIA

[d]CONRAD, Department of Obstetrics and Gynecology, Eastern Virginia Medical School, Norfolk, VA, 23507 USA

[e]Center for Targeted Drug Delivery, Department of Biomedical and Pharmaceutical Sciences, Chapman University School of Pharmacy, Harry and Diane Rinker Health Science Campus, Irvine, California 92618, USA

*Corresponding authors:

K. Parang: Center for Targeted Drug Delivery, Department of Biomedical and Pharmaceutical Sciences, Chapman University School of Pharmacy, Harry and Diane Rinker Health Science Campus, Irvine, California 92618, USA; Tel.: +1-714-516-5489; Fax: +1-714-516-5481; E-mail: parang@chapman.edu.

G. Doncel: Eastern Virginia Medical School, 601 Colley Avenue, Norfolk, Virginia, 23507, USA; Tel.: +1-757-446-8908; Fax: +1-757-446-5770; E-mail: DoncelGF@evms.edu.

H. K. Agarwal: Department of Pharmaceutical Sciences, School of Pharmacy, South University-Savannah Campus, 709 Mall Boulevard, Savannah, GA 31406, USA; Phone: 912-201-8143; Fax: 912-201-8153; E-mail: hagarwal@southuniversity.edu

Table of Contents **Page**

In vitro data

Figure S1	S3
Figure S2	S4

NMR data for selected compounds

Figure S3	S5
Figure S4	S6
Figure S5	S7
Figure S6	S8
Figure S7	S9
Figure S8	S10
Figure S9	S11
Figure S10	S12
Figure S11	S13
Figure S12	S14

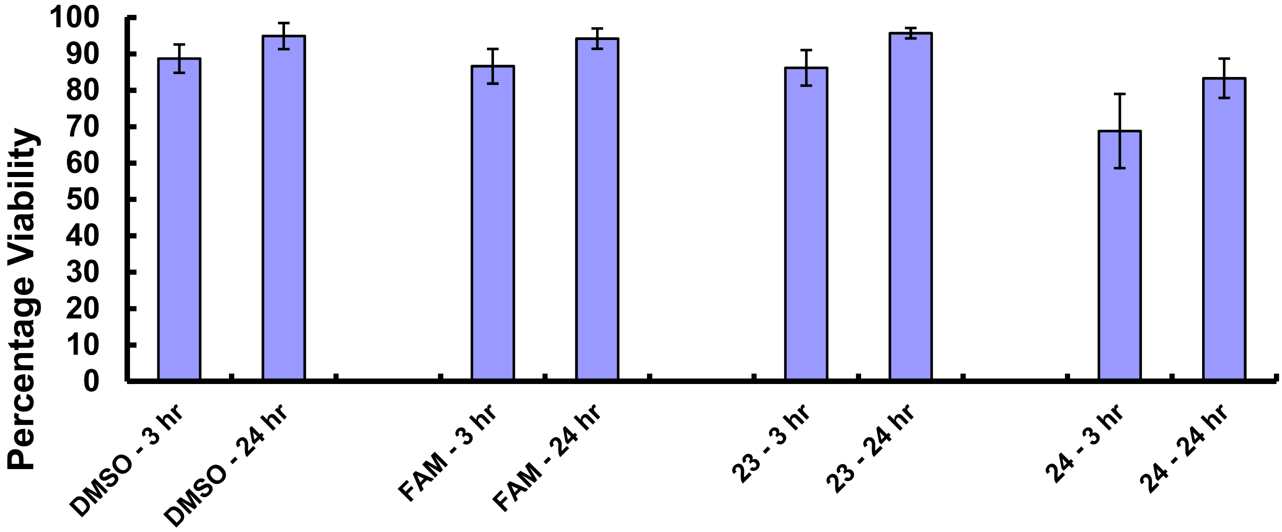

Figure S1. Cell viability assay after 3 h and 24 h incubation of **23** and **24** with CCRF-CEM cells. DMSO and FAM were used as positive controls.

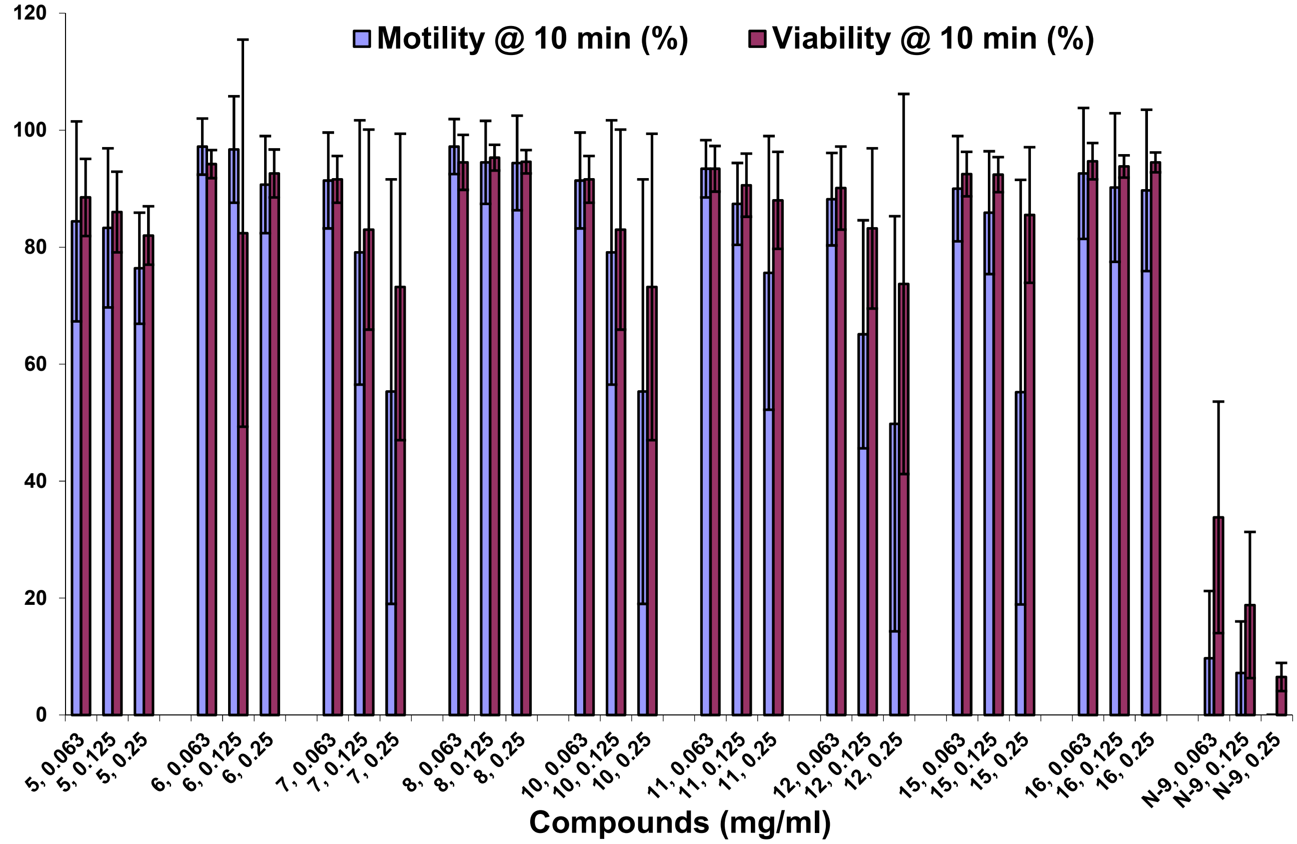

Figure S2. *In vitro* assays for spermicidal activity of fatty acyl conjugates of FLT and AZT in comparison to N-9.

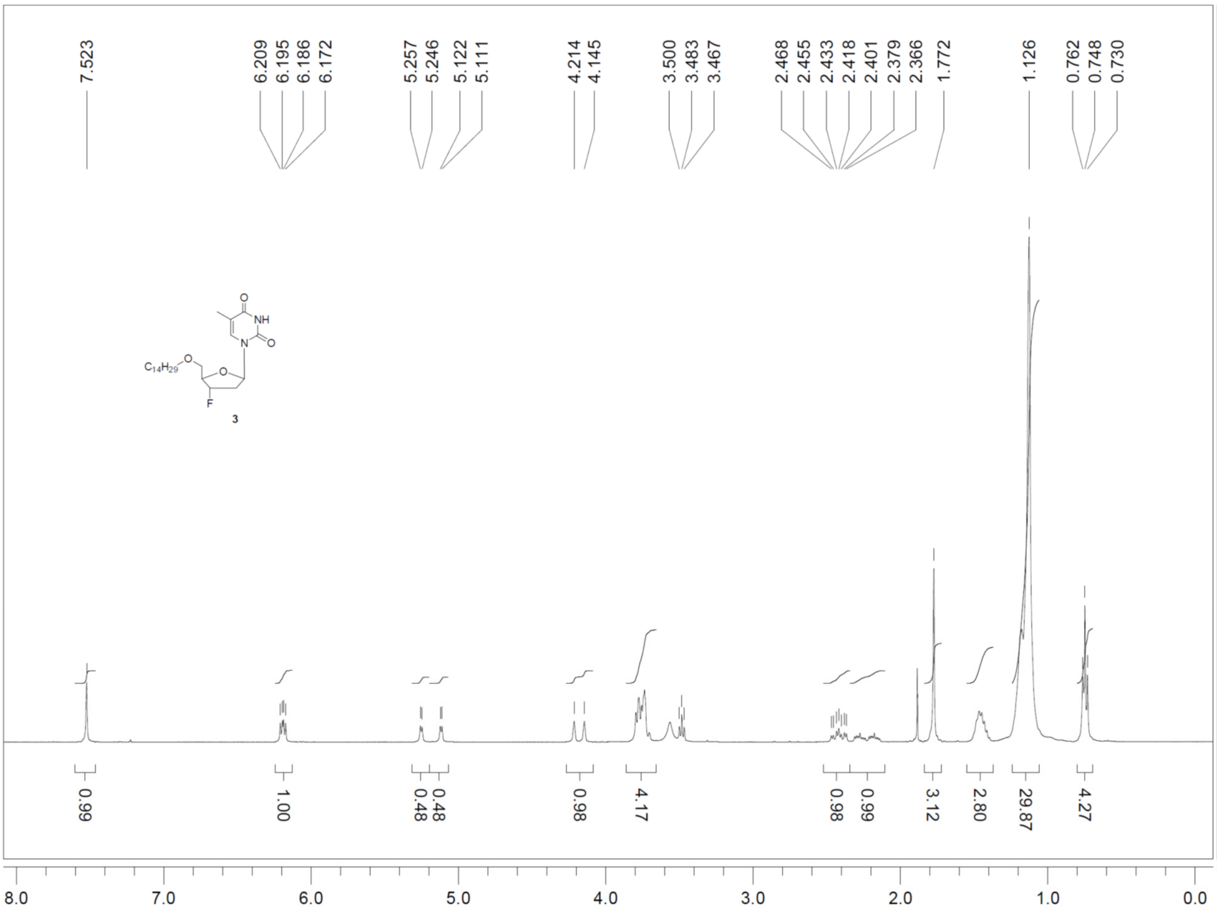

Figure S3. ^{1}H NMR of **3**.

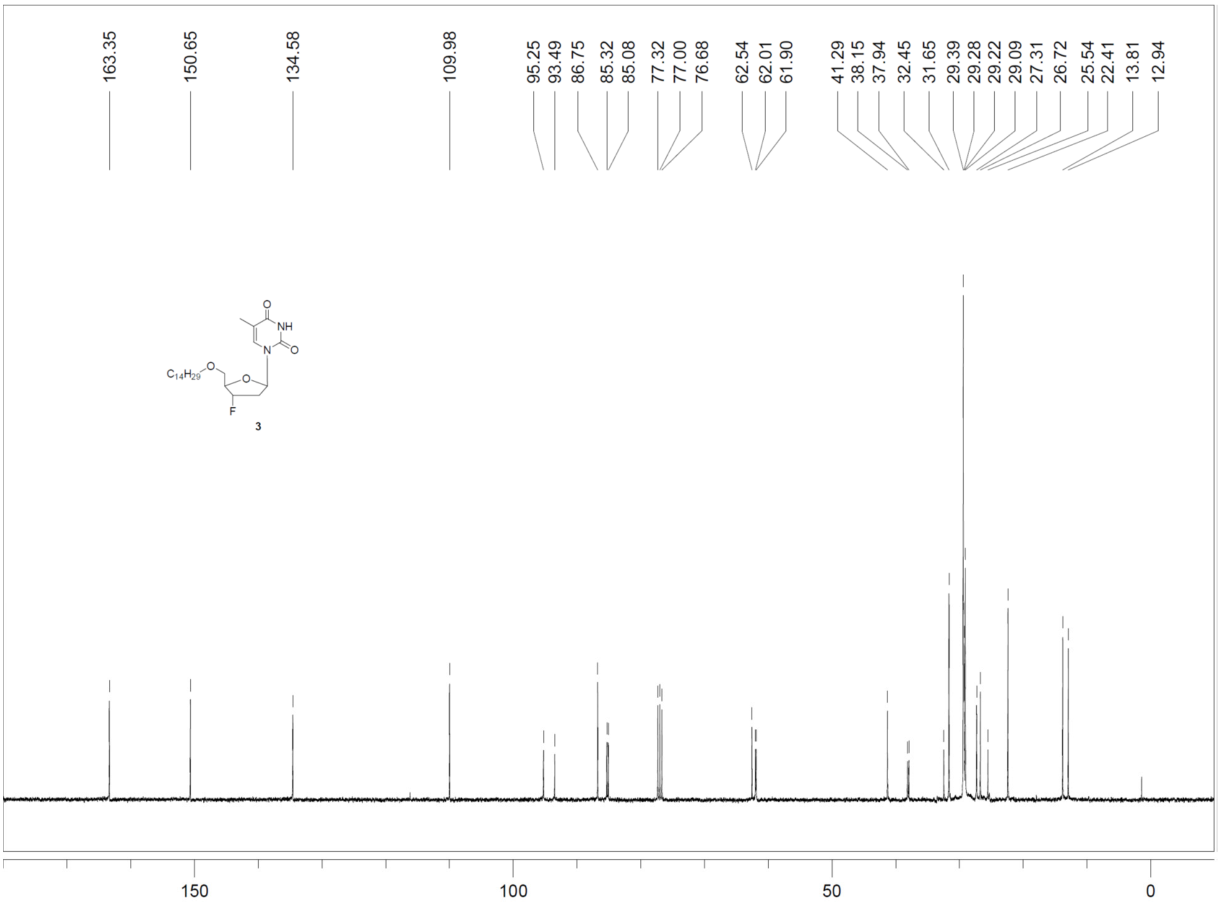

Figure S4. ^{13}C NMR of **3**.

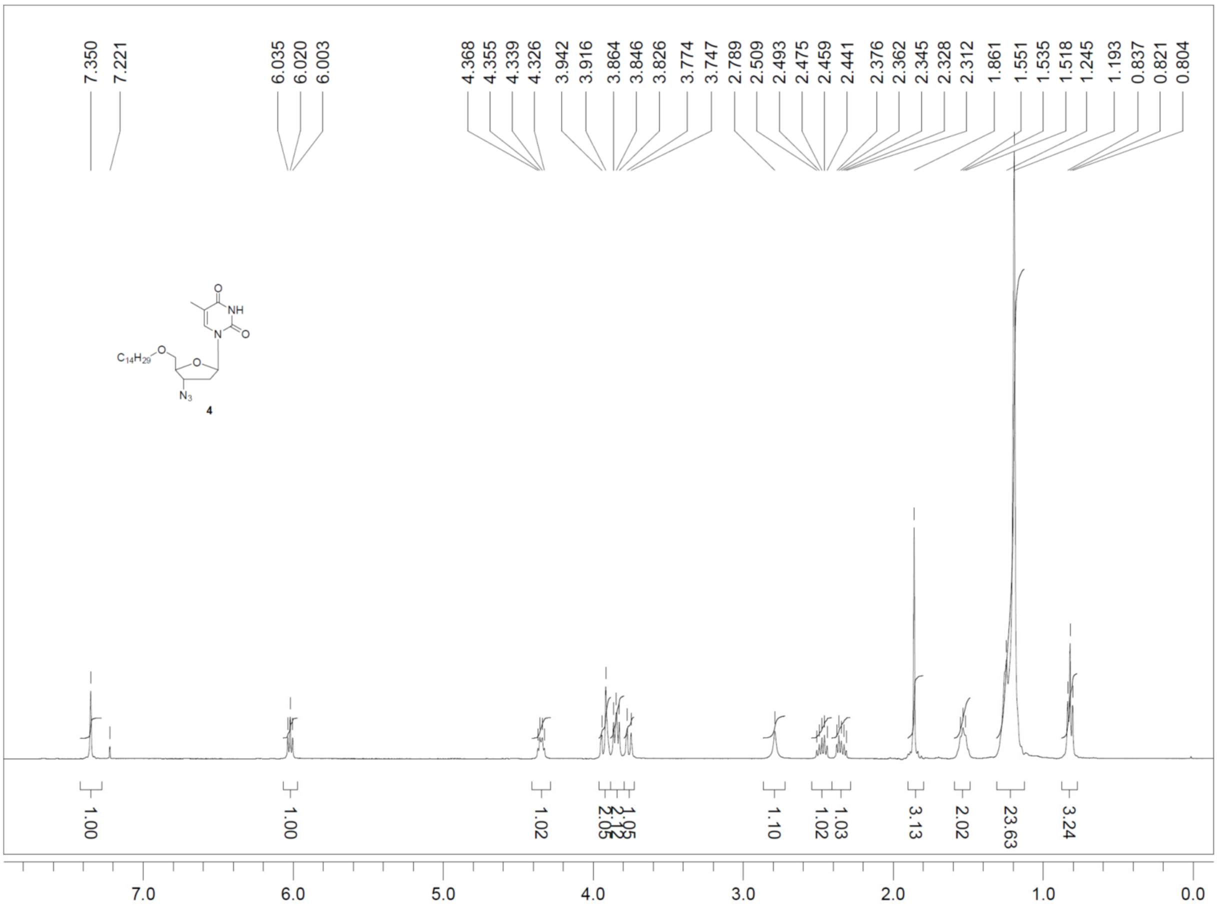

Figure S5. ^{1}H NMR of **4**.

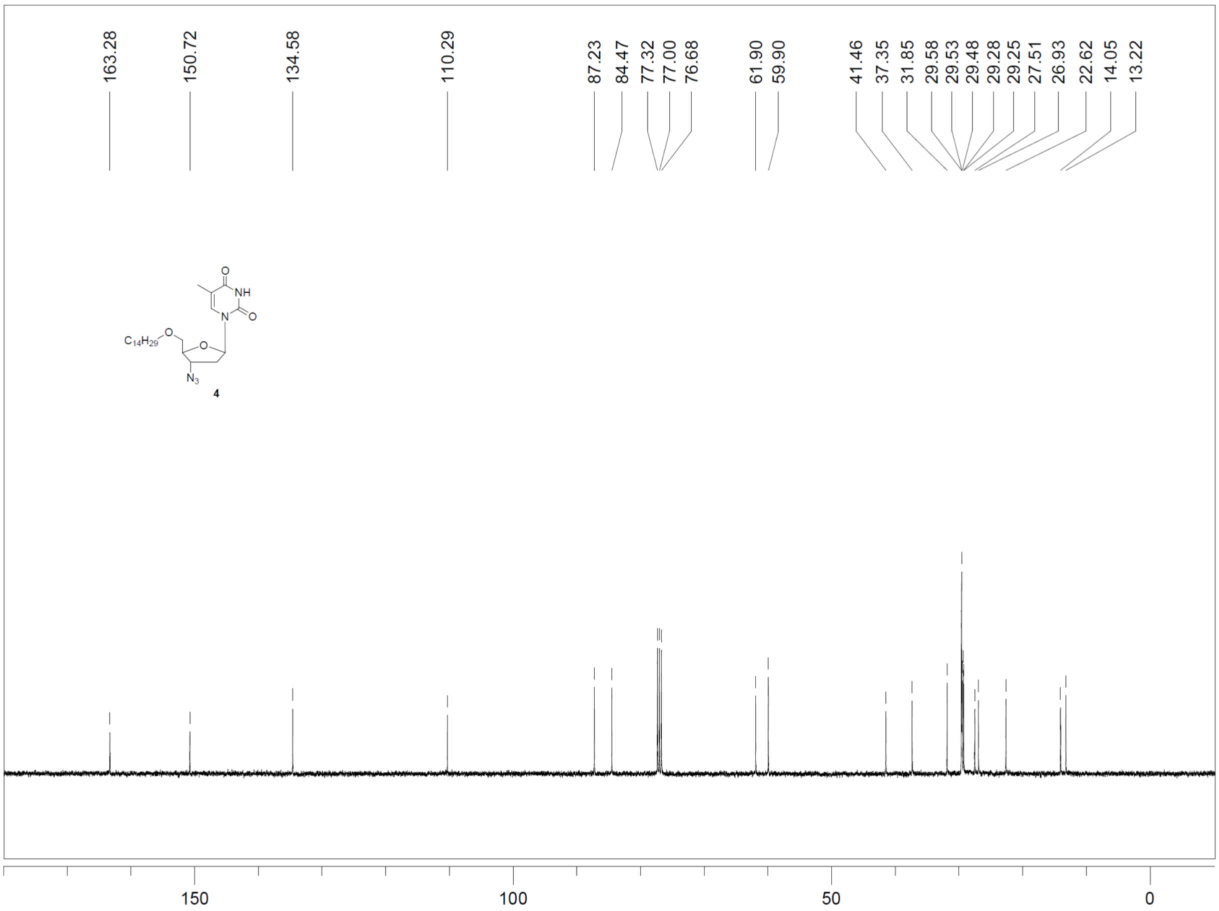

Figure S6. ^{13}C NMR of **4**.

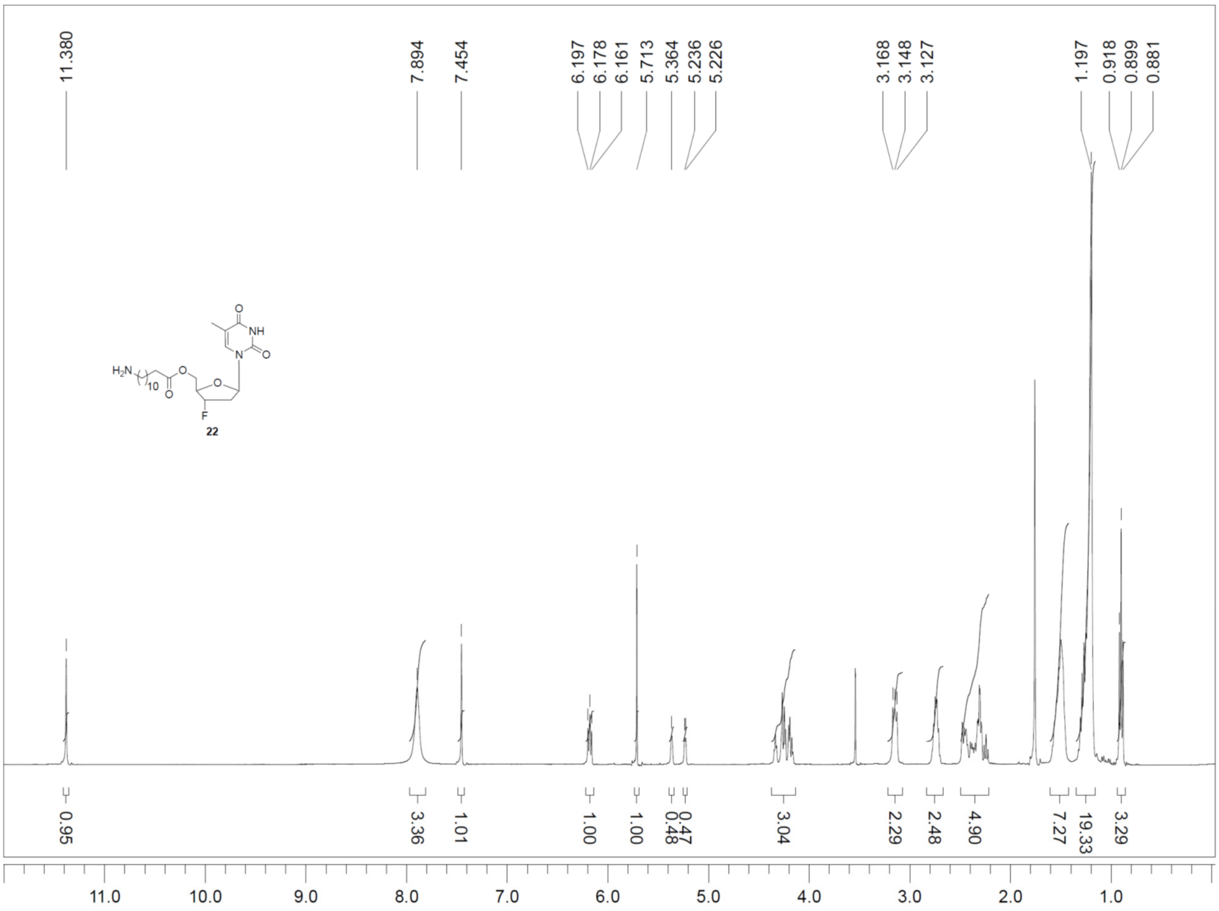

Figure S7. ^{1}H NMR of **22**.

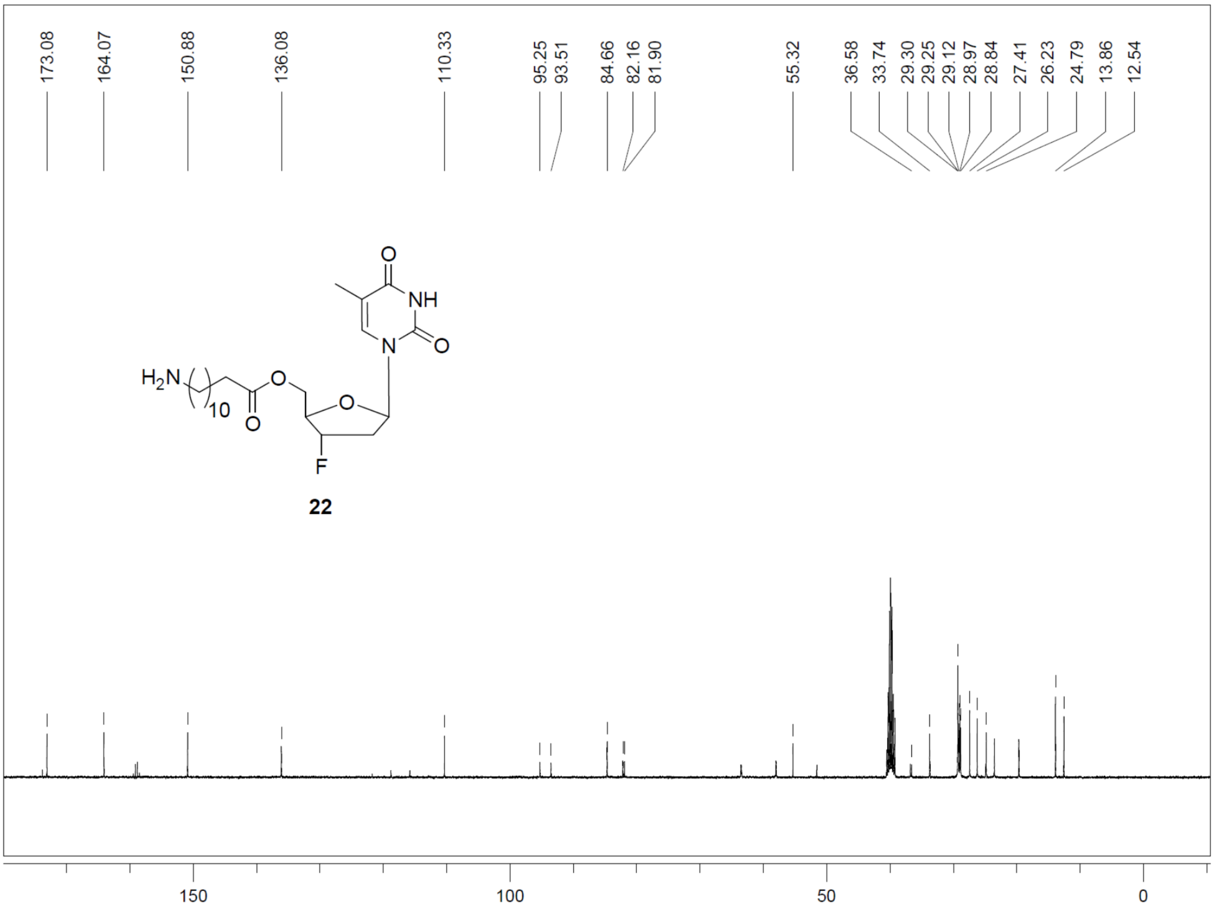

Figure S8. ^{13}C NMR of **22**.

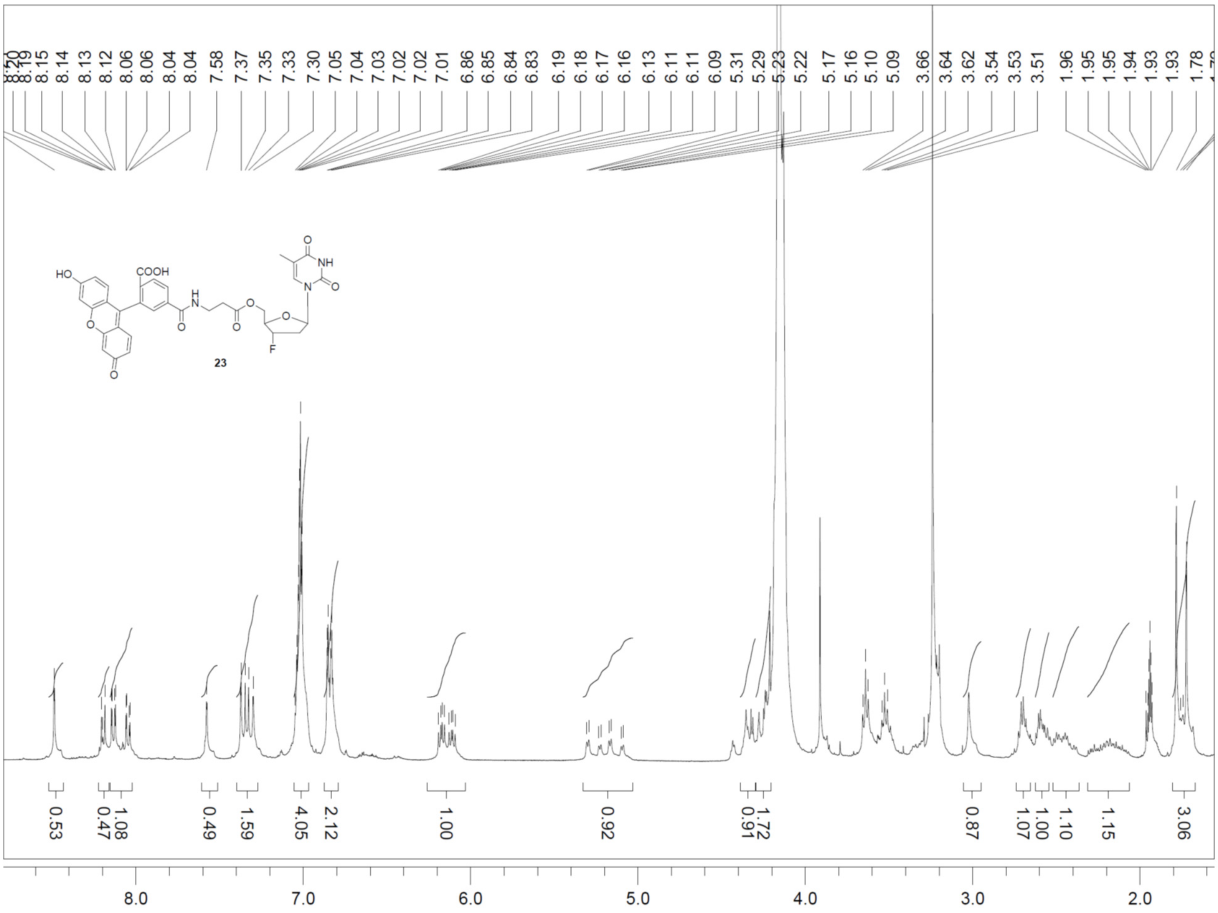

Figure S9. ^{1}H NMR of **23**.

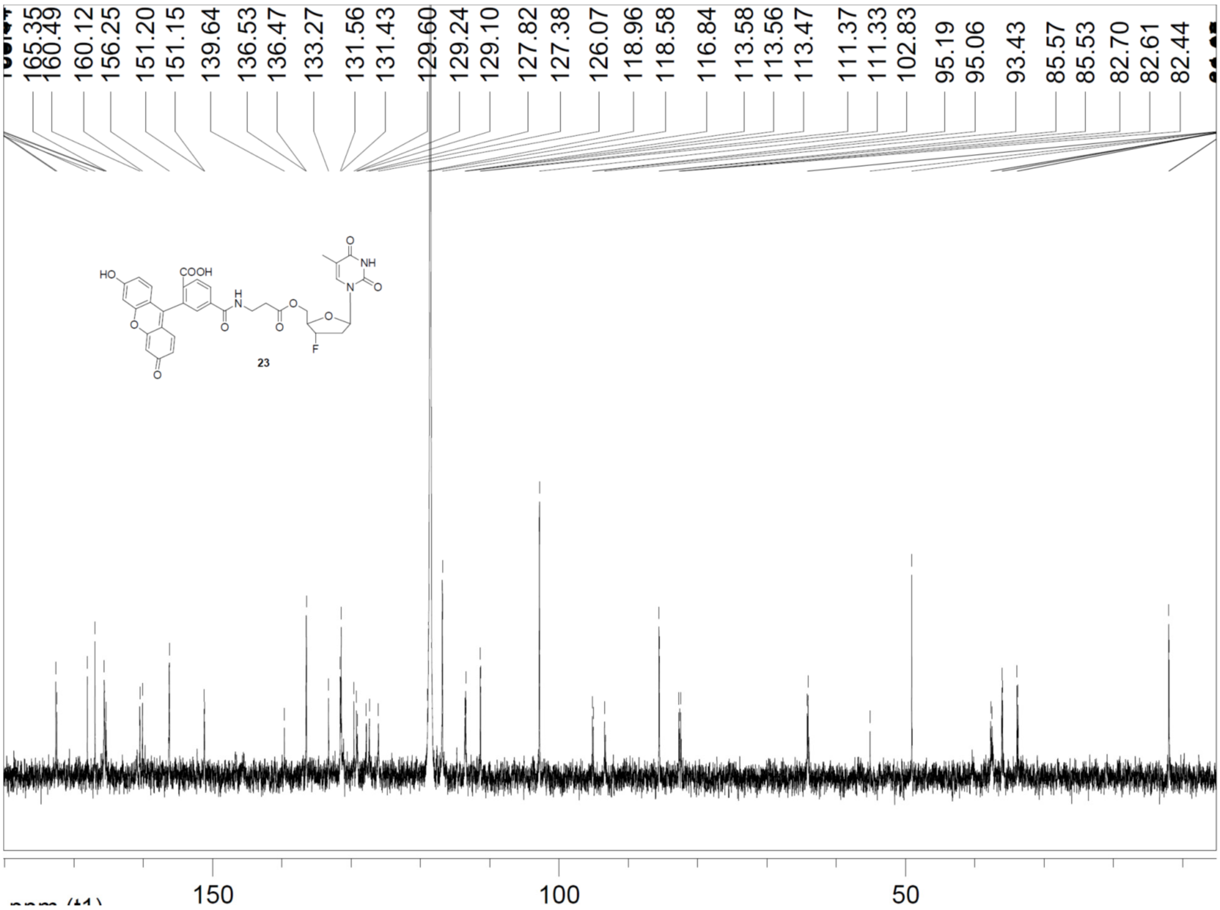

Figure S10. ^{13}C NMR of **23**.

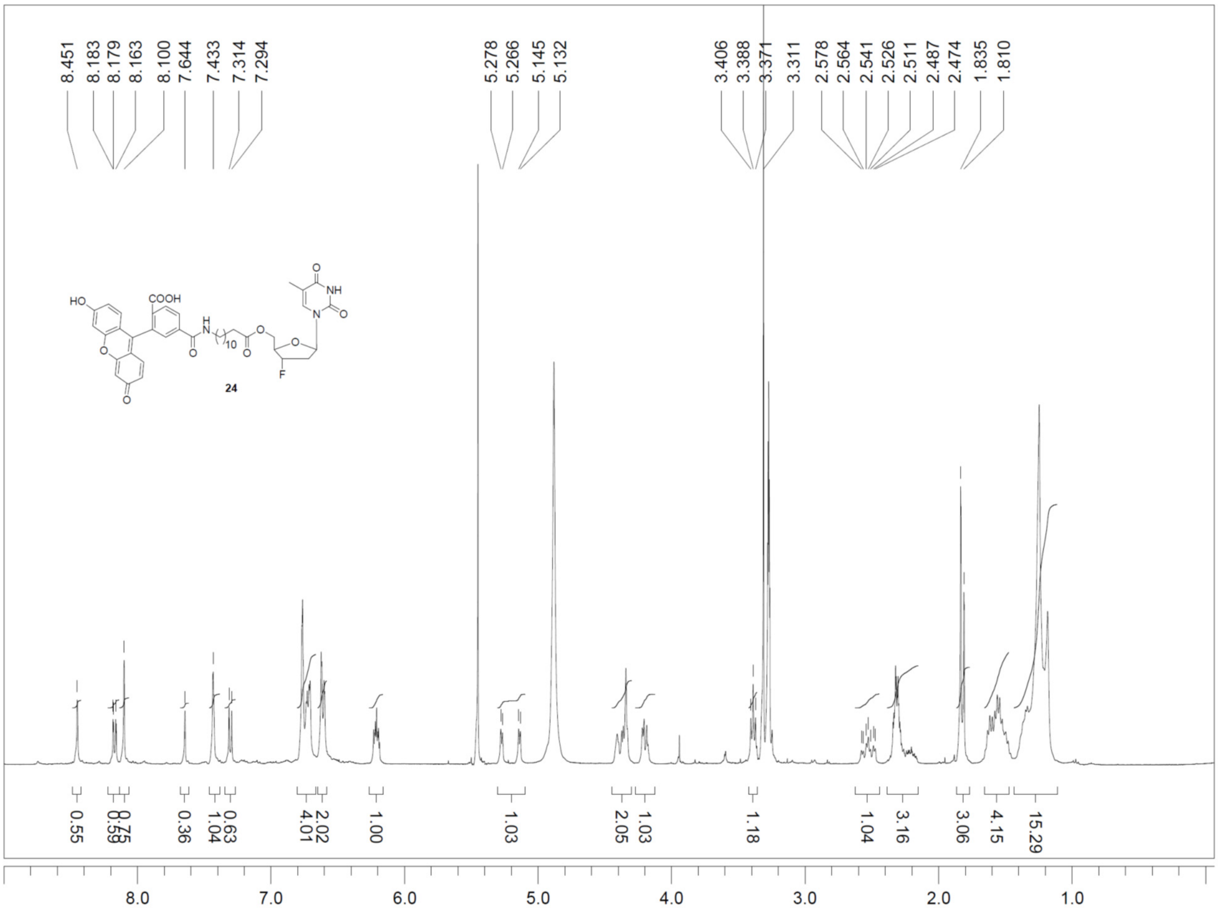

Figure S11. ^{1}H NMR of **24**.

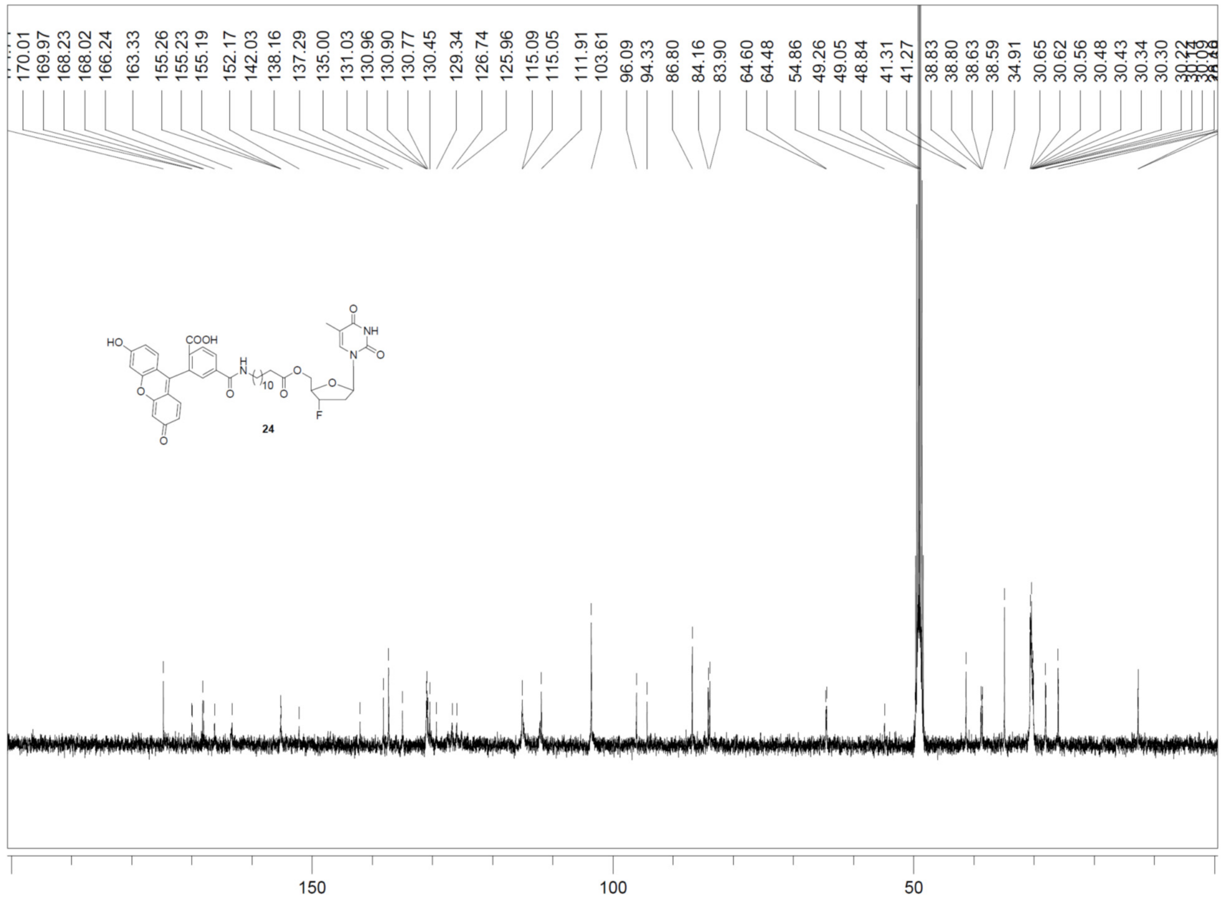

Figure S12. ^{13}C NMR of **24**.
